# Recurrent Chronic Subdural Hematoma After Burr-Hole Surgery and Postoperative Drainage: A Systematic Review and Meta-Analysis

**DOI:** 10.1227/ons.0000000000000794

**Published:** 2023-06-30

**Authors:** Roger Lodewijkx, Merijn Foppen, Kari-Anne Mariam Slot, William Peter Vandertop, Dagmar Verbaan

**Affiliations:** * Department of Neurosurgery, Neurosurgical Center Amsterdam, Amsterdam UMC Location University of Amsterdam, Amsterdam, The Netherlands;; ‡ Amsterdam Neuroscience, Amsterdam, The Netherlands

**Keywords:** Hematoma, Subdural, Chronic, Trephining, Recurrence, Mortality

## Abstract

Reported recurrence rates of chronic subdural hematoma treated by burr-hole surgery with postoperative drainage vary considerably in the literature. We performed a systematic review and meta-analysis to define the recurrence rate of burr-hole surgery with postoperative drainage.

**METHODS:**

PubMed and EMBASE were searched, and Preferred Reporting Items for Systematic Reviews and Meta-Analyses guidelines were followed. We used the Newcastle-Ottawa scale and Cochrane risk-of-bias tool for quality assessment of included studies and the random-effects model to calculate pooled incidence rates in R with the metaprop function if appropriate.

**RESULTS:**

The search yielded 2969 references; 709 were screened full text, and 189 met the inclusion criteria. In 174 studies (34 393 patients), the number of recurrences was reported as per patient and 15 studies (3078 hematomas) reported the number of recurrences per hematoma, for a pooled incidence of 11.2% (95% CI: 10.3-12.1; I^2^ = 87.7%) and 11.0% (95% CI: 8.6-13.4; I^2^ = 78.0%), respectively. The pooled incidence of 48 studies (15 298 patients) with the highest quality was 12.8% (95% CI 11.4-14.2; I^2^ = 86.1%). Treatment-related mortality (56 patients) has a pooled incidence of 0.7% (95% CI 0.0-1.4; I^2^ = 0.0%).

**CONCLUSION:**

The recurrence rate of chronic subdural hematoma treated by burr-hole surgery and postoperative drainage is 12.8%.

**ABBREVIATIONS:**
aSDH: acute subdural hematoma
cSDH: chronic subdural hematoma
I^2^: I-squared statistic
NA: not available
PI: prediction interval.

Over the past 3 decades, the incidence of chronic subdural hematoma (cSDH) has nearly tripled for patients older than 80 years, and because of the aging population, it is expected to become the most common cranial neurosurgical condition among adults by 2030.^1,2^ Although burr-hole surgery is the first treatment of choice for symptomatic cSDH,^3-5^ this procedure inherently carries a risk of a recurrent hematoma. Reported recurrence rates vary considerably (0%-33%), probably because surgical strategies also vary widely, from 1 or 2 burr holes with or without postoperative drainage to subdural or epidural location of drains.^6-8^

In recent years, less invasive, nonsurgical options to treat cSDH have been explored, such as medical treatment (steroids or tranexamic acid) or embolization of the middle meningeal artery, but randomized controlled trials are still lacking or could not prove any benefit.^9,10^ To accurately calculate how many patients need to be included in such randomized controlled trials (RCTs), sample size calculations should be based on reliable outcome measures in the control group, treated according to modern standards.

As the use of postoperative drainage has been shown to be clearly superior to burr-hole surgery without postoperative drainage,^5^ we performed a systematic review and meta-analysis to define a reliable rate of recurrence after burr-hole surgery with postoperative drainage in patients with a cSDH.

## METHODS

### Search Strategy and Study Selection

The results of this systematic review are reported in accordance with the guidelines of the Preferred Reporting Items for Systematic Reviews and Meta-Analyses checklist.^11^ This review was not registered, and a protocol was not prepared. We searched Medline (ovid) and EMBASE (embase.com) on 4th June 2021 from inception, using terms on subdural hematoma and burr hole, to identify all studies from 1946 reporting on recurrent cSDH after burr-hole surgery (see **Supplementary Table 1**, http://links.lww.com/ONS/A922, entitled “Syntax search”).

Articles with less than 25 patients, certain publication types (letter to the editor, commentary, survey, narrative review, study protocol), or records containing other language than English, German, French, Spanish, or Dutch were excluded. Systematic reviews and meta-analyses were only taken into consideration for forward and backward snowballing to identify any additional relevant articles. Records without abstracts were automatically passed into the full-text screening phase.

**FIGURE 1.**
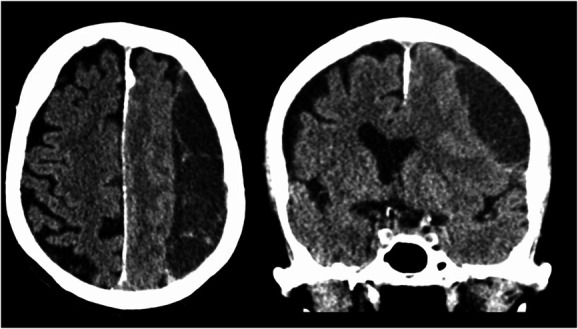
Computed tomography scan of a left-sided chronic subdural hematoma in an axial and coronal plane.

**FIGURE 2.**
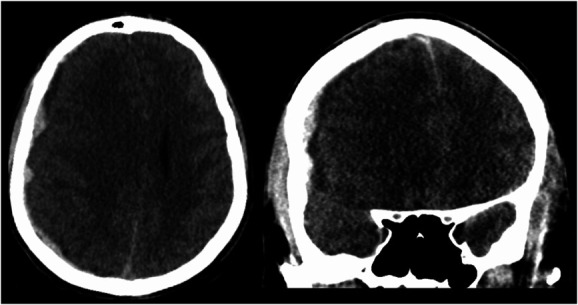
Computed tomography scan of a right-sided acute-on-chronic subdural hematoma in an axial and coronal plane.

**FIGURE 3.**
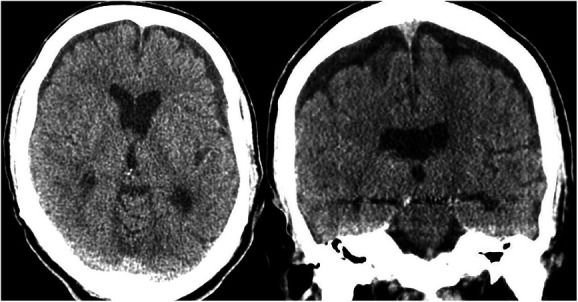
Computed tomography scan of a bilateral subdural hygroma in an axial and coronal plane.

Studies were eligible for inclusion if (1) patients were 18 years or older; (2) were diagnosed with a cSDH, acute-on-chronic subdural hematoma, or when a study described both cSDH and subdural hygroma; (3) treated with burr-hole surgery and postoperative drainage; and (4) the number of recurrences or recurrence rate was explicitly reported. In Figures 1-3, we present 3 images of a cSDH, acute-on-chronic subdural hematoma, and a subdural hygroma. Postoperative drainage had to be performed in a minimum of 95% of cases to be included. A definition of a cSDH recurrence was not mandatory for inclusion, but the study had to describe recurrences or reoperations. When a study consisted of multiple treatment groups with different strategies and the patient group of interest was distinguishable (burr-hole surgery with additional drainage), the data regarding the number of patients, recurrences, and postoperative drainage of that specific subgroup of patients were collected. In case a study described that patients did not receive a postoperative drain because of brain expansion during surgery, the study was included.

Exclusion criteria were (1) medical treatment for cSDH or embolization of the middle meningeal artery before, or after, burr-hole surgery; (2) other type of surgery than burr-hole surgery; (3) enlarged and endoscopy-assisted burr-hole surgery; and (4) reported structural cause for cSDH, such as arachnoid cysts or vascular malformations.

### Data Extraction

Two investigators (R.L. and M.F.) independently screened title and abstract to identify potential suitable records. Differences in judgment were discussed and resolved with mutual consent. Next, both investigators independently screened full texts, based on the inclusion and exclusion criteria and independently performed forward and backward snowballing of systematic reviews and meta-analyses to identify any additional relevant articles. Systematic reviews and meta-analyses were only taken into consideration for forward and backward snowballing to identify any additional relevant articles. A third rater (W.P.V.) adjudicated any discrepancies.

### Data Collection

The following preoperative characteristics were extracted: the number of patients, mean or median age, sex, antithrombotic therapy, history of trauma, Glasgow Coma Scale (GCS) score, hematoma laterality, and total number of cSDHs (bilateral hematomas counted as 2 hematomas). Regarding operative treatment the following characteristics were collected: number of burr-holes made as per standard protocol per side, number of patients receiving irrigation, type of irrigation used, number of patients receiving a postoperative drain, type of drain, number of patients not receiving a drain because of brain expansion during surgery, and postsurgical treatment. Whenever the definition of a recurrent cSDH was stated, this was noted, as well as the number of recurrences, whether the recurrence was measured per patient or per hematoma, number of recurrences treated by surgery, and number of patients in whom the recurrence was detected by computed tomography scan, or clinical symptoms. Furthermore, data on frequency and causes of mortality directly related to cSDH treatment and the occurrence of re-recurrence were collected when possible.

### Quality Assessment

The Newcastle-Ottawa scale for cohort studies, which is validated for assessing the quality of observational cohort studies, was used.^12^ According to the assigned number of stars, the following subdivision was made: 7–9, high quality; 4–6, high risk of bias; and 0–3, very high risk of bias. In case there was no nonexposed cohort in an included study, the maximum amount of stars to be assigned was 8. Thereby, the subdivision for these studies subsequently was 6–8, high quality; 3–5, high risk of bias; and 0–2, very high risk of bias. In addition, the Cochrane risk of bias for randomized controlled trials was used for studies in which a randomization method was used.^13^ Risk-of-bias judgment was determined by the overall result of the 6 domains: low risk of bias (low risk of bias for all domains), some concerns (some concerns in at least 1 domain for this result but not to be at high risk of bias for any domain), or high risk of bias (high risk of bias in at least 1 domain) accordingly. Each paper was graded and assigned a score by 2 authors [R.L. and D.V.]. A subgroup analysis was performed of the studies with the highest quality, meaning studies with Newcastle-Ottawa scale score 7–9 and score 6–8 in case of the absence of a nonexposed cohort and low risk of bias judgment determined on the Cochrane risk of bias for randomized controlled trials.

### Statistical Analysis

RStudio (R: A language and environment for statistical computing. R Foundation for Statistical Computing. URL https://www.R-project.org/) with the “meta” and “metafor” package was used to perform single-arm meta-analysis including 95% CI. To assess whether effect sizes were consistent across the included studies, heterogeneity was quantified. A *P*-value ≤.1 for the χ^2^ test of heterogeneity was considered to suggest significant heterogeneity. In addition, the I-squared (I^2^) statistic was used, which describes the percentage of variation across studies that is due to heterogeneity rather than chance. I^2^ values of 25%, 50%, and 75% would, respectively, assign adjectives of low, moderate, and high categories of heterogeneity.^14^ In case of high heterogeneity, the random-effects model was used to correctly interpret the results.^15^ Metaprop function in R was used to determine single-arm prevalence, whenever possible. In addition, for single-arm prevalence of recurrence, a forest plot was created, and 95% prediction interval (PI) was calculated with the metaprop function in case of at least 10 included studies.^16^ The prediction interval helps in the clinical interpretation of the heterogeneity by estimating what true treatment effects can be expected in future settings.^17^

Single-arm prevalence analyses of a variable consisting of either 0% or 100% incidences in the included studies was determined by dividing the total number of cases in the included studies by the total number of cases of this variable.

We assessed the presence of bias using a funnel plot. The funnel plot was created with the pooled incidence and random-effects model by plotting the recurrence rate against the standard error.

## RESULTS

### Literature Search

**FIGURE 4.**
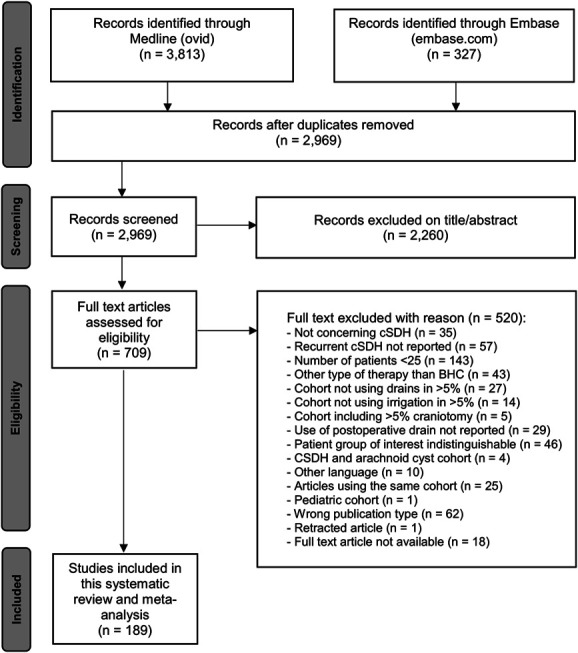
Flow chart diagram of literature search and selection.

The online search yielded a total of 4140 records. Screening the references of 33 systematic reviews yielded 15 additional articles. Duplicates were removed and yielded 2969 articles. After screening on title and abstract, 2260 records were excluded. The full texts of the remaining 709 articles were assessed for eligibility. Of the remaining full texts, 520 were excluded. In total, 189 articles reporting on 36 971 patients were selected for further analysis (Figure 4). Most studies were of a retrospective nature (n = 166) and otherwise randomized controlled trials (n = 23).

### Quality Assessment

**FIGURE 5.**
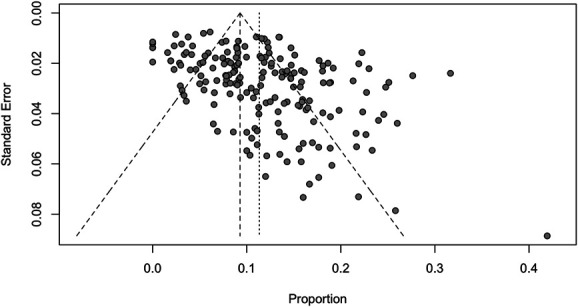
Funnel plot of recurrence rates reported in 189 included studies (36 971 patients) for evaluating bias. The plot shows that the included studies in this meta-analysis do not seem to be symmetrically ranged around the pooled incidence of recurrence, shown by the smaller dashed line. This could be due to the relatively high heterogeneity reported in this study.

The funnel plot shows that included studies do not seem to be symmetrically ranged around the pooled incidence of recurrence (Figure 5). Smaller studies seem to cause a more scattered array of recurrence rate. The risk-of-bias scores are shown in Table 1. Regarding the Newcastle-Ottawa scale and adjusted scale for cohort studies, the highest quality was observed in 50 (30.9%) studies, high risk of bias was observed in 104 (64.2%) studies, and very high risk in 8 (4.9%) studies.

**TABLE 1.** Baseline Characteristics of the 189 Included Studies

Author and year of publication	No.of patients	Mean/median age (SD/IQR/range)	Male sex (%)	No. of patients on antithrombotic medication (%)	No. of patients with a history of trauma (%)	No. of patients with a history of alcohol abuse (%)	No. of patients with unilateral cSDH (%)	No. of patients with bilateral cSDH (%)	Total no. of hematomas	No. of burr-holes performed	Irrigation method applied^b^	Postoperative drain location	Postoperative drainage time^c^	Follow-up in months	No. of patients with recurrence (%)	Quality assessment
Abboud et al,^18^ 2018	201	72 (range 25-95)	140 (70)	84 (42)	85 (42)	NA	183 (91)	18 (9)	219	1	Warm saline	Subdural	≤48 h	6	38 (18.9)	5^h^
Abouzari et al,^19^ 2007	84	56.5 (range 21-88)	59 (70)	0 (0)	0 (0)	0 (0)	84 (100)	0 (0)	84	1	Warm saline	NA	≤48 h	3	9 (10.7)	High^i^
Adachi et al,^20^ 2014	120	79	76 (63)	47 (39)	NA	23 (19)	120 (100)	0 (0)	120	1	Other	Subdural	≤24 h	6	11 (9.2)	7^h^
Adrian et al,^21^ 2017	60	69	41 (68)	6 (10)	NA	3 (5)	59 (98)	1 (2)	61	1	Warm saline	Subdural, subperiosteal	≤24 h	3	5 (5)	6^h^
Ahmed et al,^22^ 2011	25	NA	NA	NA	NA	NA	NA	NA	NA	1	Saline	Subdural	≤48 h	1	4 (16)	High^i^
Ak et al,^23^ 2017	71	NA	NA	NA	NA	NA	NA	NA	NA	2 or 3	NA	NA	NA	NA	8 (11.3)	2^h^
Amano et al,^24^ 2020^e^	323	80.0 (±8.6) and 75.5 (±11.0)	209 (65)	108 (33)	222 (69)	NA	209 (65)	114 (35)	437	1	Other	Subdural	NA	6	62 (16.5)^f^	5^h^
Aung et al,^25^ 1999	50	62 (range 54-72)	29 (58)	NA	50 (100)	NA	NA	NA	NA	2	Ringer solution/Hartmann solution	Subdural	≤48 h	3	0 (0)	4^h^
Baechli et al,^26^ 2004	354	68.3 (±17.0) (range 2-94)	228 (64)	144 (41)	272 (77)	NA	276 (78)	78 (22)	432	1 or 2	NA	Subdural	≤48 h	NA	48 (13.6)	5^h^
Bankole et al,^27^ 2011	73	NA	NA	2 (3)	30 (41)	13 (18)	51 (70)	22 (30)	95	1 or 2	Saline	Subdural	≤48 h	NA	9 (12.5)	5^g^
Bartek et al,^28^ 2017	1254	NA	NA	555 (44)	NA	NA	938 (75)	316 (25)	1570	NA	NA	Subgaleal, subperiosteal	NA	6	169 (13.5)	6^h^
Bartley et al,^29^ 2020	172	74.5 (±12) and 75.1 (±13)	127 (74)	80 (47)	NA	NA	139 (81)	33 (19)	205	1 or 2	Warm Ringer solution	Subdural	≤24 h	6	15 (8.7)	5^h^
Bellut et al,^30^ 2012	113	77 (±13) and 71 (±13)	77 (68)	62 (55)	NA	NA	83 (73)	30 (27)	143	2	Warm saline	Subdural, subperiosteal	≤48 h	3	18 (15.9)	5^h^
Blaauw et al,^31^ 2020	1029	73.5 (±11)	773 (75)	571 (55)	571 (55)	NA	772 (75)	257 (25)	1286					3	115 (11.2)	6^h^
Borger et al,^32^ 2012	322	76 (±7.9) (range 65-94)		162 (50)	233 (72)	NA	245 (76)	77 (24)	399	1	Saline	Subdural	NA	0.5	89 (22.3)^f^	4^h^
Carlisi et al,^33^ 2017	35	81.3 (6.3)	22 (63)	22 (63)	22 (63)	NA	29 (83)	6 (17)	41	NA	NA	NA	≤48 h	NA	3 (8.6)	4^h^
Carslen et al,^34^ 2011	206	NA	NA	NA	NA	NA	NA	NA	NA	1	Ringer solution/Hartmann solution	Subdural, subgaleal	≤24 h	6	29 (14.1)	5^g^
Castro-Rodriguez et al,^35^ 2016	200	88.5 (±3.2)	107 (54)	71 (36)	114 (57)	3 (2)	167 (84)	33 (17)	233	2	Saline	Subdural	≤72 h	3	26 (13)	6^h^
Certo et al,^36^ 2019	30	77.1 (range 57-87) and 76.4 (range 61-91)	19 (63)	NA	NA	NA	NA	NA	NA	1	Saline	Subdural	≤72 h	16	1 (3.3)	4^g^
Chan et al,^37^ 2017	149	74 (range 42-95)	116 (78)	46 (31)	NA	13 (9)	149 (100)	0 (0)	149	2	NA	NA	≤48 h	6	13 (8.7)	7^g^
Chang et al,^38^ 2020	122	73.6 (±11.6) (range 36-95)	83 (68)	71 (58)	73 (60)	9 (7)	79 (65)	43 (35)	165					6	14 (11.5)	7^h^
Chandran et al,^39^ 2017	52	33.7 (range 18-40)	32 (62)	2 (4)	48 (92)	13 (25)	52 (100)	0 (0)	52	1	Other	NA	NA	1	1 (1.9)	2^h^
Chen et al,^40^ 2020	171	66.0 (range 59-75)	137 (80)	22 (13)	99 (58)	NA	138 (81)	33 (19)	204					6	13 (6.4)^f^	High^i^
Cheng et al,^41^ 2014	342	77.2 (±11.4)	235 (69)	51 (15)	201 (59)	NA	269 (79)	73 (21)	415	NA	Saline	Subdural	≤120 h	3	41 (11.9)	5^h^
Choi et al,^42^ 2016	502	67 (±13)	341 (68)	NA	302 (60)	NA	NA	NA	NA	1 or 2	NA	NA	NA	NA	37 (7.4)	4^h^
Choi et al,^43^ 2020	230	69.4 (±13.1)	164 (71)	36 (16)	NA	34 (15)9	144 (63)	86 (37)	316					NA	49 (21.3)	5^h^
Chon et al,^44^ 2012	420	67.3 (range 37-92)	334 (80)	151 (36)	237 (56)	NA	352 (84)	68 (16)	488	1	NA	NA	≤72 h	3	92 (21.9)	6^h^
Choudhury et al,^45^ 1994	44	65	37 (84)	4 (9)	31 (70)	NA	37 (84)	7 (16)	51	2	Saline	Subdural	≤72 h	3	1 (2.3)	5^h^
Dobran et al,^46^ 2019	50	92.4 (range 90-100)	32 (64)	35 (70)	27 (54)	3 (6)	34 (68)	16 (32)	66					6	7 (14)	4^h^
D'Oria et al,^47^ 2020	210	66.5 (±6) and 67.2 (±7.1)	115 (55)	55 (26)	NA	13 (6)	210 (100)	0 (0)	210	1 or 2	Saline	Subdural	≤48 h	30	31 (14.8)	5^h^
Djientcheu et al,^48^ 2011	195	55 (range 21-89)	155 (79)	3 (2)	159 (82)	25 (13)	156 (80)	NA	NA	1 or 2	Saline	Subgaleal	≤48 h	9	6 (3.1)	4^h^
Dran et al,^49^ 2007	198	75 (±13) (range 33-98)	142 (72)	58 (29)	150 (76)	13 (7)	169 (85)	29 (15)	227	1	Saline	Subdural	≤48 h	17.5	16 (8)	6^h^
Drapkin,^50^ 1991	53	Range 16-97	29 (55)	5 (9)	27 (51)	NA	50 (94)	3 (6)	59	2	Saline	Subdural	Other	NA	10 (19)	5^h^
Edem et al,^51^ 2019	74	70 (range 24-96)	57 (77)	0 (0)	NA	NA	74 (100)	0 (0)	74	NA	NA	NA	NA	6	16 (21.6)	6^g^
Eggert et al,^52^ 1984	100	NA	NA	19 (19)	NA	NA	NA	NA	NA	NA	NA	NA	NA	17	11 (11)	3^h^
Eppel et al,^53^ 1999	50	67.3 and 70.3 (range 27-96)	35 (70)	9 (18)	50 (100)	8 (16)	42 (84)	8 (16)	58	1 or 2	Other	Subdural	≤120 h	28	8 (16)	5^h^
Ernestus et al,^54^ 1997	94	NA	NA	NA	NA	NA	NA	NA	NA	NA	Saline	Subdural	≤96 h	NA	17 (18.1)	4^h^
Erol et al,^55^ 2005	35	NA	NA	NA	NA	NA	32 (91)	3 (9)	38	1 or 2	Saline	Subdural	≤48 h	1	5 (14.3)	High^i^
Flint et al,^56^ 2017	659	76 (range 67-83)	464 (70)	NA	NA	NA	NA	NA	NA	1	NA	Subdural	≤48 h	6	60 (9.1)	6^g^
Flores et al,^57^ 2017	220	59	167 (76)	NA	NA	NA	220 (100)	0 (0)	220	2	Saline	Subdural	≤24 h	12	13 (5.9)	4^h^
Frati et al,^58^ 2004	35	69.4	24 (69)	NA	35 (100)	0 (0)	30 (86)	5 (14)	40	1	Saline	Subdural	≤72 h	12	5 (14.3)	4^h^
Fujisawa et al,^59^ 2021	208	74 (IQR 66-83), 74 (IQR 66-81)	153 (74)	32 (15)	130 (63)	12 (5)	NA	NA	NA					3-6	19 (9.1)	High^i^
Gabarros et al,^60^ 2000	83	66.5 (range 17-86)	59 (71)	1 (1)	NA	NA	78 (94)	5 (6)	88	2	Saline	NA	≤48 h	12	10 (12)	6^g^
Gelabert Gonzalez et al,^61^ 2005	1000	72.7 (±11.4)	628 (63)	122 (12)	617 (62)	132 (13)	903 (90)	97 (10)	1097	NA	Saline	Subdural	≤120 h	NA	61 (6.1)	4^h^
Gernsback et al,^62^ 2016	215	66	155 (72)	157 (73)	NA	NA	195 (91)	33 (15)	261	1 or 2	NA	Subdural	≤24 h	NA	16 (6.1)^f^	3^h^
Gilsbach et al,^63^ 1980	51	58.7 and 54.8	37 (73)	NA	NA	NA	45 (88)	6 (12)	57	1	Ringer solution/Hartmann solution	Subdural	NA	NA	9 (17.7)	4^h^
Glancz et al,^64^ 2019	577	78 (IQR 98-85)	394 (68)	245 (42)	361 (63)	NA	386 (67)	177 (31)	740	1 or 2	NA	Subdural, subgaleal	≤72 h	2	45 (7.8)	7^h^
Gonugunta a Buxton,^65^ 2001	184	68 and 72	86 (47)	34 (18)	NA	NA	NA	NA	NA	1	Other	Subdural	NA	6 (minimum)	27 (14.7)	4^h^
Goto et al,^66^ 2015	414	77.3	279 (67)	84 (20)	NA	NA	323 (78)	91 (22)	505					6	37 (8.9)	7^h^
Gurelik et al,^67^ 2007	42	58.4	28 (67)	NA	7 (17)	0 (0)	NA	NA	NA	1 or 2	Warm saline	Subdural	≤48 h	8	8 (19)	High^i^
Hamilton et al,^68^ 1993	29	64	NA	NA	NA	NA	NA	NA	NA	1	NA	Subdural	NA	4	3 (10.3)	3^g^
Han et al,^69^ 2009	180	62.2 (±14.3)	131 (73)	NA	180 (100)	NA	155 (86)	25 (14)	205	1 or 2	Warm saline	Subdural	≤120 h	1 (minimal)	10 (5.6)	5^h^
Hani et al,^70^ 2019	361	72.6 (±11.0) + 74.8 (±11.0)	244 (68)	191 (53)	NA	NA	NA	NA	NA	2	NA	Subdural, subgaleal	≤48 h	6	83 (23)	6^h^
Harders et al,^71^ 1982	100	61.6 and 62.9	70 (70)	19 (19)	62 (62)	7 (7)	92 (92)	8 (8)	108	2	Ringer solution/Hartmann's solution	Subdural	Other	17	24 (24)	3^h^
Hennig & Kloster,^72^ 1999	90	NA	NA	NA	NA	NA	82 (91)	8 (9)	98	2	Gentamicin-induced irrigation	Subdural	≤48 h	NA	7 (7.1)^f^	3^g^
Heringer et al,^73^ 2017	96	NA	NA	NA	NA	NA	74 (77)	22 (23)	96	1 or 2	Saline	Subdural	NA	7.6	15 (15.6)	5^g^
Hirai et al,^74^ 2021	320	77.3 (±10.9)	228 (71)	94 (29)	NA	NA	274 (86)	46 (14)	366					3	37 (10.6)^f^	8^h^
Hori et al,^75^ 2018	92	81.9 (±8.5) and 77.5 (±8.9)	63 (68)	25 (27)	61 (66)	1 (1)	77 (84)	15 (16)	107	NA	NA	Subdural	NA	3	15 (16.3)	6^h^
Hsieh et al,^76^ 2016	75	71.9 (±12.5)	53 (71)	20 (27)	38 (51)	19 (25)	52 (69)	23 (31)	98	1	Saline	Subdural	NA	8 (mean)	7 (9.3)	5^h^
Huang et al,^77^ 2013	98	69.3 (±12.8)	81 (83)	15 (15)	73 (74)	14 (14)	73 (74)	25 (26)	123	1 or 2	Other	Subdural	NA	10.6 (mean)	14 (14.3)	5^h^
Huang et al,^78^ 2020	140	68.7 (±12.7)	114 (81)		91 (65)	NA	NA	NA	NA	1	NA	Subdural	≤48 h	3	12 (8.6)	4^h^
Iftikar et al,^79^ 2016	34	66.7 (±14.0)	28 (82)	NA	NA	NA	NA	NA	NA	NA	Saline	Subdural	≤48 h	1-10 (min-max)	6 (17.6)	6^g^
Ishfaq,^80^ 2017	62	72 (range 55-85)	41 (66)	NA	33 (53)	NA	52 (84)	10 (16)	72	1 or 2	NA	Subdural, subgaleal	≤96 h	NA	7 (11.3)	High^i^
Ishibashi et al,^81^ 2011	34	79.1 (10)	19 (56)	5 (15)	NA	NA	31 (91)	3 (9)	36	1	Warm saline	Subdural	≤48 h	NA	1 (2.9)	High^i^
Jang et al,^82^ 2015	30	68 (range 58-78.5)	21 (70)	5 (17)	NA	6 (20)	NA	NA	NA	2	Saline	Subdural	NA	3	3 (10)	9^g^
Jang et al,^83^ 2020	291	71.8 (±11.8)	208 (71)	93 (32)	NA	125 (43)	175 (60)	116 (40)	407	1 or 2	Saline	NA	≤72 h	2	29 (10)	7^h^
Janowski & Kunert,^84^ 2012	45	66 (range 24-86)	30 (67)	NA	NA	NA	42 (93)	3 (7)	48	NA	NA	Subdural	≤24 h	NA	7 (15.6)	5^h^
Jeong et al,^85^ 2014	125	69.4 (±12.3)	92 (74)	35 (28)	81 (65)	NA	96 (77)	29 (23)	154	NA	Warm saline	NA	NA	3	8 (6.4)	3^h^
Jukovic et al,^86^ 2014	35	NA	NA	NA	NA	NA	NA	NA	NA	NA	Warm saline	Subdural	≤48 h	6	0 (0)	5^g^
Jung et al,^87^ 2015	182	68.1	131 (72)	46 (25)	125 (69)	65 (36)	147 (81)	35 (19)	217	1 or 2	Ringer solution/Hartmann solution	Subdural	≤72 h	12	25 (13.7)	7^h^
Kale et al,^88^ 2017	90	55.61 (±18.7)	54 (60)	12 (13)	75 (83)	NA	75 (83)	15 (17)	105	1 or 2	Saline	Subdural	≤96 h	6	9 (8.6)^f^	5^h^
Kaliaperumal et al,^7^ 2012	50	Range 17-91	33 (66)	25 (50)	29 (58)	NA	42 (84)	8 (16)	58	2	Warm saline	Subdural, subperiosteal	≤48 h	6	0 (0)	High^i^
Kang et al,^89^ 2007	302		236 (78)	NA	NA	NA	267 (88)	35 (12)	337		NA	NA	≤48 h	3	24 (7.9)	4^h^
Kaminogo et al,^90^ 1999	38	69.3 (±12.0)	34 (89)	NA	25 (66)	NA	32 (84)	6 (16)	44	1	Saline	Subdural	≤24 h	6	4 (9.1)^f^	5^h^
Kanyi et al,^91^ 2018	119	61.3 (range 19-94)	95 (80)	0 (0)	65 (55)	41 (34)	95 (80)	23 (19)	141	2	Saline	Subdural	≤48 h	0.5	6 (5)	4^h^
Kareem & Adams,^92^ 2018	36	79 (range 55-95)	26 (72)	24 (67)	34 (94)	NA	30 (83)	6 (17)	42	1	Warm Ringer solution	Subperiosteal	≤48 h	3	4 (11)	3^h^
Katayama et al,^93^ 2018	88	75.8 (±9.5)	67 (76)	10 (11)	NA	NA	77 (88)	11 (13)	99	NA	Saline	Subdural	≤24 h	3	11 (12.5)	High^i^
Khan et al,^94^ 2019	60	62 (±13.7) (range 38-94)	40 (67)	0 (0)	NA	NA	53 (88)	7 (12)	67	1 or 2	Other	Subdural	NA	NA	14 (23.3)	High^i^
Kim et al,^95^ 2011	259	63.7 (±16.9)	191 (74)	NA	167 (64)	15 (6)	NA	NA	NA	1 or 2	NA	NA	≤72 h	6	23 (8.9)	3^g^
Kim et al,^96^ 2014	114	67.6 (±11.5)	88 (77)	NA	51 (45)	NA	114 (100)	0 (0)	114	NA	Saline	NA	≤72 h	3	28 (24.6)	6^g^
Kim et al,^97^ 2016	100	70.4 (±12.6)	62 (62)	36 (36)	100 (100)	NA	NA	NA	NA	1	Saline	NA	≤48 h	6	26 (26)	9^g^
Kim,^98^ 2017	246	68.6 (±12.2)	173 (70)	59 (24)	187 (76)	NA	183 (74)	63 (26)	309	1 or 2	NA	Subdural	NA	6	31 (12.6)	7^h^
Kiymaz et al,^99^ 2007	29	62.7 (±3.2)	24 (83)	NA	18 (62)	2 (7)	NA	NA	NA	2	Warm saline	Subdural	≤96 h	NA	2 (6.9)	4^g^
Klein et al,^100^ 2021	407	74.7 (±13.2) and 76.6 (±8.2)	280 (69)	209 (51)	NA	NA	317 (78)	90 (22)	497					32.7 ± 16.1	72 (17.7)	7^h^
Kocaman & Yilmaz,^101^ 2019	30	78 (range 64-92)	20 (67)	NA	NA	NA	30 (100)	0 (0)	30	2	Warm saline	Subdural	NA	6	5 (16.7)	4^h^
Kotwica & Brzezinski,^102^ 1991	131	Range 18-82	101 (77)	0 (0)	93 (71)	45 (34)	NA	NA	NA	2	Saline	Subdural	≤48 h	24 (minimum)	3 (2.3)	3^h^
Kristof et al,^103^ 2008	75	73	38 (51)	NA	48 (64)	NA	58 (77)	17 (23)	92	NA	Ringer solution/Hartmann solution	Subdural	≤96 h	NA	17 (22.7)	6^g^
Krupp & Jans,^104^ 1995	214	65 (±15)	128 (60)	13 (6)	28 (13)	15 (7)	167 (78)	45 (21)	257	1 or 2	Other	Subdural	≤72 h	NA	53 (25)	5^h^
Kurabe et al,^105^ 2010	182	77.3 (range 65-98)	123 (68)	38 (21)	NA	40 (22)	148 (81)	34 (19)	216	1	NA	Subdural	≤48 h	3	14 (7.7)	5^h^
Kuroki et al,^106^ 2001	45	67.3 (range 43-88)	32 (71)	NA	NA	NA	40 (89)	5 (11)	50	1	Saline	Subdural	≤120 h	44	5 (11.1)	6^h^
Kutty & Johny,^107^ 2014	70	NA	55 (79)	25 (36)	NA	NA	53 (76)	17 (24)	87	1	Saline	Subdural	≤72 h	3	2 (2.9)	High^i^
Kwon et al,^108^ 2000	145	59.3 (range 23-89)	104 (72)	NA	NA	NA	115 (79)	30 (21)	175	1	Saline	Subdural	≤120 h	6	6 (4.1)	5^h^
Lee et al,^109^ 2004	38	70	25 (66)	NA	NA	6 (16)	NA	NA	NA	2	Saline	Subdural	≤72 h	NA	6 (16)	6^g^
Lee et al,^110^ 2009	32	65.3 (±12.1)	25 (78)	NA	NA	NA	NA	NA	NA	2	Saline	NA	≤120 h	NA	7 (22)	5^g^
Lee & Park,^111^ 2014	114	77.9	81 (71)	30 (26)	70 (61)	33 (29)	86 (75)	28 (25)	142	NA	Saline	Subdural	≤72 h	3	19 (16.7)	5^h^
Lee et al,^112^ 2018	131	68 (±17)	85 (65)	35 (27)	71 (54)	NA	NA	NA	NA	1	Warm saline	Subdural	≤72 h	6	7 (5.3)	5^h^
Lepic et al,^113^ 2021	55	72.8 (±11.5)	37 (67)	NA	NA	NA	36 (65)	19 (35)	67					1	3 (5.5)	3^h^
Leung et al,^114^ 2001	46	NA	NA	NA	NA	NA	NA	NA	NA	NA	Saline	NA	NA	NA	3 (6.5)	6^h^
Liliang et al,^115^ 2002	75	65.3 (range 16-92)	63 (84)	NA	53 (71)	7 (9)	59 (79)	16 (21)	91	1 or 2	Saline	NA	≤48 h	30	3 (4)	6^h^
Li et al,^116^ 2017	115	68.3 (±5.1)	76 (66)	16 (14)	NA	NA	103 (90)	24 (21)	NA					6	11 (9.6)	6^h^
Lin,^117^ 2011	270	62.3 (±24.5)	218 (81)	NA	NA	NA	NA	NA	NA	1	Saline	Subdural	≤72 h	NA	32 (11.9)	6^g^
Liu et al,^118^ 2010	398	58.1 (±18.1)	338 (85)	6 (2)	275 (69)	12 (3)	304 (76)	94 (24)	492	1	Saline	NA	≤120 h	NA	15 (3.8)	3^h^
Liu et al,^119^ 2019	328	65.1 (±13.8) (range 22-93)	281 (86)	17 (5)	170 (52)	NA	267 (81)	61 (19)	389	1	Other	NA	≤48 h	6	8 (2.4)	8^h^
Lo et al,^120^ 2013	98	69.3 (±12.8) (range 29-93)	81 (83)	15 (15)	73 (74)	14 (14)	73 (74)	25 (26)	123	1 or 2	Saline	Subdural	NA	3	14 (14.3)	5^h^
Lu et al,^121^ 2018	87	68.4 (±6.5) (range 55-82)	71 (82)	NA	72 (83)	NA	87 (100)	0 (0)	87	2	Warm saline	Subdural	NA	6-12	4 (4.6)	5^h^
Maldaner et al,^122^ 2019	253	75	190 (75)	106 (42)	NA	NA	180 (71)	73 (29)	326	2	NA	Subperiosteal	≤48 h	3	40 (15.8)	7^h^
Markwalder,^123^ 2000	32	61.4	22 (69)	NA	26 (81)	NA	27 (84)	5 (16)	37	2	NA	Subdural	≤48 h	1.5	1 (3.1)	3^h^
Martinez Perez et al,^124^ 2020	90	76.4 (±11.2)	71 (79)	42 (47)	NA	NA	78 (87)	12 (13)	102	2	NA	Subdural	≤48 h	6	17 (18.9)	8^h^
Mellergard & Wisten,^125^ 1996	218	70.5 (range 11-93)	155 (71)	22 (10)	135 (62)	32 (15)	193 (89)	25 (11)	243	1	Saline	Subdural	≤48 h	1.5	30 (12.3)^f^	4^h^
Mersha et al,^126^ 2020	195	57.6	137 (70)	NA	124 (64)	NA	147 (75)	48 (25)	243	1	Warm saline	Subdural	≤24 h	4	13 (6.6)	6^h^
Mezue et al,^127^ 2011	116	NA	NA	26 (22)	91 (78)	NA	NA	NA	NA	1 or 2	NA	NA	NA	NA	9 (7.8)	3^h^
Miah et al,^128^ 2020	60	73 (range 34-95)	49 (82)	31 (52)	45 (75)	NA	39 (65)	21 (35)	81	1 or 2	Warm Ringer solution	Subdural	≤48 h	6	13 (22)	7^g^
Miki et al,^129^ 2019	277	78.6 (±11.1)	181 (65)	70 (25)	193 (70)	NA	234 (84)	43 (16)	320	1	Other	Subdural	≤48 h	3	50 (18.1)	7^h^
Missori et al,^130^ 2000	31	38 (range 20-50)	24 (77)	NA	24 (77)	2 (6)	27 (87)	4 (13)	35	1	Other	NA	≤24 h	2	2 (6)	4^h^
Morales-Gomez et al,^131^ 2020	155	65.9 (±16.6) (range 18-95)	127 (82)	8 (5)	101 (65)	14 (9)	124 (80)	31 (20)	186					2	18 (11.6)	5^h^
Mori & Maeda,^132^ 2001	500	67.3 (±15.3) and 71.3 (±14.2)	359 (72)	26 (5)	286 (57)	32 (6)	412 (82)	88 (18)	588	2	Saline	Subdural	≤48 h	3	49 (9.8)	4^h^
Motoie et al,^133^ 2018	787	79 (IQR 72-85)	559 (71)	199 (25)	NA	195 (daily alcohol consumption)^d^ (25)	671 (85)	116 (15)	903	NA	Other	Subdural	NA	NA	96 (12.2)	6^h^
Munoz-Bendix et al,^134^ 2017	112	NA	63 (56)	63 (56)	NA	NA	88 (79)	24 (21)	136					1	25 (22.3)	3^h^
Nakagawa et al,^135^ 2019	381	NA	NA	NA	NA	NA	NA	NA	NA	1	Saline	Subdural	≤24 h	6	71 (18.6)	4^h^
Nakaguchi et al,^136^ 2001	106	67	82 (77)	0 (0)	63 (59)	NA	86 (81)	20 (19)	126	1	Saline	NA	≤48 h	3	21 (17)^f^	5^h^
Nunta-Aree et al,^137^ 2017	75	60.7 (66 median)	79 (hematoma's) (NA)	35 (47)	31 (41)	2 (3)	48 (64)	27 (36)	102	2	Other	NA	NA	2	10 (13.3)	4^h^
Okano et al,^138^ 2014	448	71.1 (range 19-97)	314 (70)	18 (4)	225 (50)	NA	344 (77)	104 (23)	552	1	Saline	Subdural	NA	42	40 (8.9)	7^h^
Oral et al,^139^ 2015	78	68.1 and 66.1 (median)	57 (73)	9 (12)	58 (74)	NA	73 (94)	5 (6)	83	1 or 2	Warm saline	Subdural, subgaleal	≤72 h	3	4 (5.1)	5^h^
Penchet et al,^140^ 1998	236	71.7	146 (62)	57 (24)	182 (77)	18 (8)	195 (83)	41 (17)	276	1 or 2	Saline	NA	≤48 h	3	10 (4.2)	3^h^
Piotrowski & Krombholz-Reindl,^141^ 1996	200	67.5	150 (75)	45 (23)	109 (55)	6 (3)	163 (82)	37 (19)	237	1	NA	Subdural	NA		16 (8)	3^h^
Poulsen et al,^142^ 2014	177	NA	NA	NA	NA	NA	NA	NA	NA	1	Warm Ringer solution	Subdural, subperiosteal	NA	3	28 (15.8)	High^i^
Qian et al,^143^ 2017	242	66.3 (±10.9) (range 36-93)	148 (61)	54 (22)	145 (60)	79 (33)	242 (100)	0 (0)	242	1	Warm Ringer solution	Subdural	≤120 h	6	39 (16.1)	7^h^
Raghavan et al,^144^ 2020	153	72.2 (±13.1)	93 (61)	41 (27)	82 (54)	26 (17)	105 (69)	48 (31)	201	1	NA	Subdural	NA	1	24 (15.7)	6^g^
Ram et al,^145^ 1993	37	70.8 (±10)	25 (68)	NA	NA	NA	33 (89)	4 (11)	41	2	Saline	Subdural	≤48 h	1	5 (13.5)	High^i^
Regan et al,^146^ 2015	61	72 (range 52-98)	36 (59)	29 (48)	38 (62)	9 (15)	NA	NA	80	1 or 2 or 3	Other	Subdural	NA	NA	4 (6.6)	5^g^
Ridwan et al,^147^ 2019	197	NA	NA	NA	NA	NA	NA	NA	NA	1 or 2	NA	Subdural	≤72 h	2	37 (18.8)	6^g^
Rohde et al,^148^ 2002	376	64	242 (64)	NA	NA	NA	NA	NA	NA	1	Ringer solution/Hartmann solution	Subdural	NA	NA	119 (31.6)	5^h^
Rovlias et al,^149^ 2015	986	69 (range 29-96)	650 (66)	237 (24)	503 (51)	132 (13)	907 (92)	79 (8)	1065	2	Other	Subdural	Other	3	117 (11.9)	6^h^
Ryu et al,^150^ 2018	187	67 (range 22-93)	135 (72)	76 (41)	93 (50)	NA	187 (100)	0 (0)	187	1	Saline	Subdural	≤48 h	3	22 (11.8)	6^h^
Sah & Rawal,^151^ 2018	102	NA	NA	NA	NA	NA	80 (78)	22 (22)	124	NA	NA	Subdural	NA	NA	9 (8.8)	5^g^
Santarius et al,^5^ 2009	108	74.4 (46-94)	83 (77)	49 (45)	NA	NA	87 (81)	21 (19)	129	2	Warm Ringer solution	Subdural	≤48 h	6	10 (9)	High^i^
Sarnvivad et al,^152^ 2011	97	60.5 (±20.1)	66 (68)	21 (22)	NA	NA	76 (78)	21 (22)	118	1	Saline	NA	NA	NA	15 (16)	4^g^
Schoedel et al,^153^ 2016	697	70.1 (range 1-97)	461 (66)	226 (32)	317 (45)	NA	NA	NA	NA	NA	NA	Subdural	NA	NA	155 (22.2)	5^g^
Schwarz et al,^154^ 2015	193	71.4 (±13.6)	137 (71)	87 (45)	143 (74)	9 (5)	138 (72)	55 (28)	250	NA	Other	Subdural	≤24 h	3	35 (18.1)	8^h^
ShafiqAlam et al,^155^ 2017	428	NA	NA	NA	NA	NA	NA	NA	NA	NA	Saline	NA	NA	NA	53 (12.4)	5^h^
Shah et al,^156^ 2014	25	NA	15 (60)	NA	NA	NA	NA	NA	NA	1	Warm saline	Subdural	≤48 h	6	3 (12)	High^i^
Shen et al,^157^ 2019	457	68.8 (range 23-92)	376 (82)	28 (6)	235 (51)	NA	311 (68)	146 (32)	603	1	Saline	Subdural	≤72 h	3	69 (15.1)	8^h^
Shim et al,^158^ 2019	60	74.5 (range 67-90)	44 (73)	0 (0)	30 (50)	NA	NA	NA	NA	1	Saline	Subdural	NA	NA	8 (13.3)	5^g^
Singh et al,^159^ 2011	52	61.2	47 (90)	NA	NA	NA	52 (100)	0 (0)	52	1	Gentamicin-induced irrigation	Subdural	NA	1	1 (2)	High^i^
Singh et al,^160^ 2014	100	NA	NA	24 (24)	72 (72)	34 (34)	87 (87)	13 (13)	NA	1 or 2	Saline	Subdural	≤48 h	6	9 (9)	High^i^
Sjavik et al,^161^ 2017	1260	73 (±11) and 74 (±13) and 74 (±13)	878 (70)	222 (18)	NA	NA	1005 (80)	217 (17)	1439	1	Gentamicin-induced irrigation	Subdural, subgaleal	≤24 h	6	169 (13.4)	6^h^
Song et al,^162^ 2014	97	70 (range 15-93)	64 (66)	NA	61 (63)	NA	94 (97)	3 (3)	100	1	Other	Subdural	≤48 h	3	16 (16.5)	6^h^
Sousa et al,^163^ 2013	778	64.3 (±15.9) (range 14-93)	643 (83)	NA	470 (60)	NA	604 (78)	174 (22)	952	1	Saline	Subdural	≤48 h	3 (minimum)	42 (5.4)	5^h^
Stanisic et al,^164^ 2013	107	72.1 (±12.8)	72 (67)	NA	NA	NA	84 (79)	23 (21)	130	1	Saline	Subdural	≤24 h	7	17 (15.9)	7^h^
Sucu & Akar,^165^ 2014	119	65.7 (±16.9)	NA	NA	NA	NA	84 (71)	35 (29)	154	1 or 2	NA	NA	≤48 h	1	4 (3.4)	2^h^
Suzuki et al,^166^ 1998	67	NA	NA	NA	NA	NA	NA	NA	NA	1	Warm saline	Subdural	≤72 h	NA	2 (3)	4^g^
Tagle et al,^167^ 2003	100	77 (±13)	77 (77)	21 (21)	43 (43)	NA	84 (84)	16 (16)	116	1 or 2	Other	NA	≤72 h	66	13 (13)	2^h^
Takei et al,^168^ 2021	277	82 (range 76-89) and 87 (range 76-92)	211 (76)	79 (29)	NA	NA	247 (89)	30 (11)	307					3	35 (11.4)^f^	8^h^
Tahsim-Oglou et al,^169^ 2012	247	75 (±13) and 77 (±8)	165 (67)	NA	NA	NA	193 (78)	54 (22)	281	2	Warm Ringer solution	Subdural	≤48 h	1	62 (25.1)	7^h^
Tailor et al,^170^ 2017	123	75.6	88 (72)	30 (24)	NA	NA	97 (79)	26 (21)	149	1 or 2	NA	Subdural	NA	6	10 (8.1)	4^g^
Tanikawa et al,^171^ 2001	33	69.3 (±14.9)	NA	NA	NA	NA	NA	NA	NA	2	Saline	Subdural	≤72 h	6	4 (12.1)	High^i^
Taussky et al,^172^ 2008	76	69 (±12) (range 38-89)	54 (71)	53 (70)	NA	NA	55 (72)	21 (28)	97	1 or 2	Ringer solution/Hartmann solution	NA	≤48 h	1	13 (13.4)^f^	5^h^
Thavara et al,^173^ 2019	63	61.4 (±13.2)	48 (76)	9 (14)	31 (49)	6 (10)	63 (100)	0 (0)	63	1	Saline	Subdural	≤24 h	NA	1 (1.6)	5^g^
Toi et al,^174^ 2019	342	77 (65-80) and 78 (65-79)	248 (73)	57 (17)	223 (65)	NA	342 (100)	0 (0)	342	1	Other	Subdural	Other	3	39 (11.4)	High^i^
Tomita et al,^175^ 2018	102	77.2 (±10.7)	66 (65)	21 (21)	76 (75)	4 (4)	89 (87)	13 (13)	115	1	Saline	Subdural	NA	NA	8 (7.8)	4^h^
Tommiska et al,^176^ 2019	71	78 (range 57- 93)	50 (70)	48 (68)	62 (87)	NA	55 (77)	15 (21)	85	1	Warm Ringer solution	Subdural	≤48 h	6	4 (5.6)	7^g^
Torihashi et al,^177^ 2008	337	75.5 (range 28-96)	228 (68)	24 (7)	81 (24)	NA	268 (80)	69 (20)	406	1	Saline	Subdural	NA	1	61 (18.1)	7^h^
Tosaka et al,^178^ 2015	46	NA	NA	NA	NA	NA	NA	NA	46	NA	Saline	Subdural	NA	1.5	5 (10.9)^f^	4^g^
Tsai et al,^179^ 2010	129	71 (range 22-97)	100 (78)	11 (9)	90 (70)	6 (5)	84 (65)	45 (35)	174	2	Saline	Subdural	NA	NA	12 (9.3)	4^h^
Tugcu et al,^180^ 2014	292	61.9 (±17.8) (range 1-96)	200 (68)	56 (19)	160 (55)	4 (1)	210 (72)	82 (28)	374	2	Warm saline	NA	≤96 h	1	43 (14.7)	7^h^
Vasella et al,^181^ 2018	28	70.4 (±16.1)	24 (86)	NA	NA	NA	19 (68)	9 (32)	37	2	Warm saline	Subdural, subperiosteal	≤48 h	9	1 (3.6)	2^h^
Wan et al,^182^ 2017	31	73.6 (range 61-83)	18 (58)	1 (3)	26 (84)	NA	31 (100)	0 (0)	NA	1	Saline	Subdural	≤120 h	3	13 (41.9)	4^g^
Wang et al,^183^ 2016	53	66.7 (±13.1)	44 (83)	0 (0)			45 (85)	8 (15)	61	1	Saline	Subdural	≤72 h	1	9 (17)	5^g^
Wang et al,^184^ 2017	45	67.3 (±12.9)	38 (84)	6 (13)	26 (58)	0 (0)	45 (100)	0 (0)	45	1	Saline	Subdural	Other	3	5 (11.1)	6^g^
Wang et al,^185^ 2017 (1)	88	65.5 (±7.8)	53 (60)	NA	NA	NA	88 (100)	0 (0)	88	1	Warm saline	NA	≤72 h	12	6 (6.8)	5^g^
Wang et al,^186^ 2017 (2)	57	64.6	52 (91)	0 (0)	47 (82)	NA	NA	NA	NA	1	Warm saline	Subdural	≤72 h	6	0 (0)	6^g^
Wang et al,^187^ 2020	653	72 (IQR 64-80)	561 (86)	NA	NA	234 (current drinking)^d^ (36)	504 (77)	149 (23)	802					3	96 (14.7)	8^h^
Weigel et al,^188^ 2015	93	75.6 (±12.9) and 72.6 (±13)	NA	0 (0)	NA	NA	73 (78)	20 (22)	113	1 or 2	Other	Subdural	≤72 h	3	13 (11.5)^f^	5^h^
Weng et al,^189^ 2019	190	68 (range 27-86)	118 (62)	NA	161 (85)	NA	190 (100)	0 (0)	190	1	Other	Subdural	NA	6	17 (8.9)	4^h^
Won et al,^190^ 2021	176	75 (range 65-80) and 74.5 (range 62-80)	126 (72)	87 (49)	NA	NA	120 (68)	56 (32)	232					3	40 (22.7)	High^i^
Wu et al,^191^ 2020	331	NA	285 (86)	22 (6)	239 (72)	95 (29)	268 (81)	63 (19)	394					3	30 (9.1)	6^h^
Yadav et al,^192^ 2016	140	53 (±22.1) (range 18-75)	101 (72)	NA	140 (100)	NA	140 (100)	0 (0)	140	1	Warm saline	Subgaleal	NA	21	5 (3.6)	6^g^
Yagnick et al,^193^ 2019	60	64.3	51 (85)	21 (35)	20 (33)	NA	42 (70)	18 (30)	78	2	Other	Subdural	NA	8	0 (0)	5^h^
Yamada et al,^194^ 2018	1080	72.1 (±14.3) (range 13-101)	711 (66)	124 (11)	827 (77)	5 (0)	883 (82)	197 (18)	1227	1	Other	Subdural	≤48 h	NA	119 (11)	5^h^
Yamada & Notori,^195^ 2020	193	78.8 (±0.8) and 78.2 (±9.8) and 79.2 (±8.7)	160 (83)	72 (37)	NA	48 (25)	85 (44)	108 (56)	232	1	Other	Subdural	≤24 h	3	16 (6.9)^f^	High^i^
Yamamoto et al,^196^ 2003	105	71.4 (±9.6) and 71.4 (±10.5)	73 (70)	4 (4)	78 (74)	41 (39)	82 (78)	23 (22)	128	1 or 2	Warm saline	NA	≤48 h	6	11 (10.5)	7^h^
Yan et al,^197^ 2017	52	66.4 (±9.4)	37 (71)	12 (23)	NA	NA	41 (79)	11 (21)	63	1	Saline	Subdural	≤72 h	12	7 (13.7)	4^g^
Yan et al,^198^ 2018	231	NA	188 (81)	0 (0)	0 (0)	NA	201 (87)	30 (13)	261	NA	Other	NA	≤48 h	3	33 (14.3)	5^h^
You et al,^199^ 2018	226	65.1 (±13.4)	184 (81)	14 (6)	161 (71)	NA	160 (71)	66 (29)	292	1 or 2	Saline	Subdural	≤48 h	12	34 (15)	8^h^
Yu et al,^200^ 2009	97	67 (range 14-93)	82 (85)	NA	NA	11 (11)	74 (76)	24 (25)	121	1	Saline	Subdural	Other	3	8 (8.2)	5^h^
Zakaraia et al,^201^ 2008	40	59.7	29 (73)	0 (0)	NA	NA	NA	NA	NA	2	Saline	Subdural	≤72 h	6	4 (10)	High^i^
Zhang et al,^202^ 2018	31	75.1	22 (71)	13 (42)	24 (77)	9 (29)	29 (94)	2 (6)	33	2	Warm saline	Subdural	NA	6	8 (25.1)	6^g^
Zhang et al,^203^ 2019	570	71 (IQR 62-79)	422 (74)	123 (22)	380 (67)	52 (9)	333 (58)	237 (42)	807	1 or 2	Gentamicin-induced irrigation	Subdural, subperiosteal	≤48 h	6	70 (12.3)	7^h^
Zumofen et al,^204^ 2009	147	71.5 (range 42-93)	113 (77)	41 (28)	117 (80)	12 (8)	111 (76)	36 (24)	183	2	Saline	Subperiosteal	≤48 h	3	22 (15)	5^h^
Pooled incidence (95% CI, I^2^)	—	—	71.9% (70.6-73.2; 84.3%)	23.6% (21.3-26.0; 98.5%)	64.2% (55.1-73.3; 99.8%)	12.3% (10.4-14.1; 95.8%)	—	—	—	^a^	See Table 5	See Table 6	See Table 6	—	See Results section	See Results section

CSF, cerebrospinal fluid; cSDH, chronic subdural hematoma; I^2^, I-squared statistic; NA, not available.

a A single burr-hole per side was made in 55.8%, a double burr-hole per side in 19.3% of cases, and in 24.9% it was reported that either a single burr-hole or a double burr- hole was used, and these could not be separated.

b Labeled according to 6 categories we established: Saline, warm saline, Ringer solution/Hartmann solution, warm Ringer solution, Gentamicin-induced irrigation and other. Category “saline” was abbreviated, full name: saline/normal saline/isotonic saline/physiological saline. “other” included: unknown (described in included studies as: irrigation/washout/rinsing), normal saline or artificial CSF, artificial CSF, or irrigation with ARTCEREB.

c Labeled according to 6 categories we established: ≤24 h, ≤48 h, ≤72 h, ≤96 h, ≤120 h, and other. “Other” included different drainage periods described in studies: 24–144 h, 192 ± 96 h, 48–144 h, 8 h until <5 mL per hour draining volume, and 24–216 h.

d Excluded in calculating history of alcohol abuse proportion.

e Only 71 patients with a bilateral cSDH underwent operation.

f Study which calculates the recurrence rate as proportion of the total number of hematomas.

g Quality assessment performed using the Newcastle-Ottawa scale.

h Quality assessment performed using the adjusted Newcastle-Ottawa scale.

i Quality assessment performed using the Cochrane risk-of-bias tool for randomized trials. High means high risk of bias.

### Recurrence Rate and Mortality

Baseline characteristics of the 189 included studies reporting the number of recurrences are shown in Table 1. The pooled incidence of 174 studies with 34 393 patients reporting 4208 recurrences per number of patients was 11.2% (95% CI: 10.3-12.1; I^2^ = 87.7%; 95% PI: 0.0-22.0). In addition, the pooled incidence of 15 studies with 3078 hematomas reporting 370 recurrences per number of hematomas was 11.0% (95% CI: 8.6-13.4; I^2^ = 78.0%, 95% PI: 2.0-20.0). Forest plots are shown in the **Supplementary Fig. 1**, http://links.lww.com/ONS/A923 and **Supplementary Fig. 2**, http://links.lww.com/ONS/A924.

The pooled incidence of reported recurrence rates at 3 months (25 articles) was 12.7% (95% CI: 10.7-14.7; I^2^ = 86.1%) and 9.8% (95% CI: 7.6-11.9; I^2^ = 89.0%) at 6 months (21 articles). The pooled incidence of recurrence in patients treated by 1 burr-hole was 12.2% and in patients treated by 2 burr-holes was 12.8%.

Pooled mortality incidences are displayed in Table 2. Mortality related to cSDH treatment was seen in 56 patients with a pooled incidence of 0.7% (95% CI 0.0-1.4; I^2^ = 0.0%) (Table 3).

**TABLE 2.** Pooled Incidence of Mortality per Time Period

Mortality period	Number of studies	Number of patients	Pooled incidence (95% CI; I^2^)
In-hospital mortality	39	188	1.3% (0.9-1.7; 78.9%)
30-d mortality	29	229	2.5% (1.7-3.2; 86.6%)
3-mo mortality	9	256	3.6% (1.9-5.3; 90.0%)
6-mo mortality	15	493	6.5% (3.6-9.4; 97.2%)
Mortality without time period indication	45	199	2.0% (1.4-2.5; 73.6%)

I^2^, I-squared statistic.

All calculated with random-effects model.

**TABLE 3.** Studies Reporting Mortality Causes Related to cSDH Treatment

Author and year	Mortality cause(s) related to cSDH treatment (number of cases)
Bartley et al,^29^ 2020	Intracerebral hematoma (n = 1), basal ganglion infarction (n = 1)
Bellut et al,^30^ 2012	Postoperative intraparenchymal hematoma (n = 1)
Carlisi et al,^33^ 2017	Postoperative (n = 1)
Chen et al,^40^ 2020	Recurrent cSDH (n = 2)
Choi et al,^42^ 2016	After taking anticoagulants (n = 3)
Djientcheu et al,^48^ 2011	Brain herniation (n = 1), inhalation pneumonia in comatose patients with delayed treatment (n = 3)
Eppel et al,^53^ 1999	Postoperative brain hemorrhage (n = 1)
Gabarros et al,^60^ 2000	Recurrent bleeding (n = 1)
Hennig et al,^72^ 1999	Infection (n = 1), rebleed after first operation (n = 1)
Kotwica et al,^102^ 1991	Large ischemic stroke of the hemisphere compressed by the hematoma (n = 1), purulent meningitis, followed by subdural empyema (n = 1)
Lepic et al,^113^ 2021	Sequence of rebleeding episodes and clinical worsening (n = 1)
Liu et al,^119^ 2019	Recurrence of cSDH for which consent for repeat surgery was not given (n = 1)
Mellergard et al,^125^ 1996	Subdural empyema after third recurrence operation (n = 1)
Mezue et al,^127^ 2011	Secondary infection (n = 1)
Missori et al,^130^ 2000	Acute subdural hemorrhage (n = 1)
Morales-Gomez et al,^131^ 2020	aSDH (n = 1), parenchymal hematoma (n = 1)
Penchet et al,^140^ 1998	Empyema (n = 1), aSDH (n = 1)
Piotrowski et al,^141^ 1996	Cerebral decompensation which were comatose before operation (n = 6)
Ridwan et al,^147^ 2019	Cerebrovascular accidents (n = 2)
Rohde et al,^148^ 2002	Intracerebral bleeding (n = 4)
Schoedel et al,^153^ 2016	Acute secondary hemorrhage (n = 5)
Shen et al,^157^ 2019	Spontaneous ventricular hemorrhage after operation (n = 1), postoperative acute epidural hemorrhage (n = 1)
Sucu et al,^165^ 2014	Subdural empyema (n = 1), aSDH (n = 1)
Suzuki et al,^166^ 1998	Extreme disturbance of consciousness did not improve postoperatively and pneumonia supervened (n = 1)
Tagle et al,^167^ 2003	Recurrence of cSDH (n = 1)
Tsai et al,^179^ 2010	aSDH (n = 2)
Zhang et al,^203^ 2019	Because of removal of the drain after which aSDH developed, the patient died 45 d later in the hospital because of pneumonia (n = 1)
Zumofen et al,^204^ 2009	Continued to bleed acutely from subdural membranes despite trepanation, refrained from second intervention (n = 2)

aSDH, acute subdural hematoma; cSDH, chronic subdural hematoma.

### Treatment Modalities

Pooled incidences and recurrence rates per irrigation method, type of postoperative drainage, and drainage time are shown in Tables 4-6, respectively. Ringer solution (also titled Hartmann solution) showed a recurrence rate of 21.4%, and irrigation fluid at room temperature (22 °C) showed a recurrence rate of 12.0%.

**TABLE 4.** Pooled Incidence of Irrigation Method

Irrigation method	No.of studies	No. of patients	Pooled incidence	Recurrence rate	Recurrence rate combined (95%CI; I^2^)^b^
Saline/normal saline/isotonic saline/physiological saline	75	13.364	47.8%	11.1%	12.0%
Ringer solution/Hartmann solution	9	1292	4.6%	21.4%	
Gentamicin-induced irrigation method	4	1972	7.0%	12.5%	
Warm saline	28	2893	10.4%	9.7%	11.5%
Warm Ringer solution	8	1113	4.0%	15.7%	
Other^a^	28	7313	26.2%	Not determined	Not determined

I^2^, I-squared statistic.

a Other includes Hartmann solution with gentamicin, normal saline or artificial CSF, artificial CSF, irrigation with ARTCEREB and those describing only irrigation/washout/rinsing.

b Saline/normal saline/isotonic saline/physiological saline and Ringer solution/Hartmann solution are combined to determine the recurrence rate, and warm saline and warm Ringer solution are combined to investigate the effect of normal vs warm irrigation method.

Calculated by dividing the number of patient per group by the total number of patients in which the irrigation method was described.

**TABLE 5.** Pooled Incidence and Recurrence Rate per Type of Postoperative Drain Location Used

Location of postoperative drain	No. of studies^a^	No. of patients	Pooled incidence	Recurrence rate^b^
Subdural	139	24 965	80.9%	12.8%
Subperiosteal	7	780	2.5%	13.6%
Subgaleal	8	1596	5.2%	9.9%
Other	13	3536	11.4%	Not determined

a In 9 studies, more than 2 drain locations were reported and could be separated per location and was calculated additionally.

b In 42 studies of which drain location was unknown, the first author was emailed to retrieve the drain location. Hereby, we determined 9 drain locations extra.

Calculated by dividing the number of patients per group by the total number of patients in which postoperative drain location was described.

“Other” includes studies reporting 2 or more drain locations which could not be separated.

**TABLE 6.** Pooled Incidence of Postoperative Drainage Time

Drainage time in hours^a^	No. of studies	No. of patients	Pooled incidence	Recurrence rate
≤24 h	17	4577	16.7%	11.4%
≤48 h	57	12 619	46.0%	11.2%
≤72 h	28	5500	20.0%	13.3%
≤96 h	5	657	2.4%	14.5%
≤120 h	11	2465	9.0%	8.3%
Other^b^	6	1623	5.9%	Not determined

a When a drainage time range was given in a study, the maximum drainage time was accounted for and calculated with in the corresponding variable.

b Other includes different drainage time periods described in studies: 24–144 h, 192 ± 96 h, 48–144 h, 8 h until <5 mL per hour draining volume, and 24–216 h.

Calculated by dividing the number of patients per group by the total number of patients in which postoperative drainage time was described.

### Subgroup Analyses

We performed a subgroup analysis of the 50 studies with the highest quality regarding risk-of-bias judgment, of which 48 (15 298 patients) described the number of recurrences (2019 recurrences) as per the patient, and 2 studies (597 patients) described the recurrences (72 hematomas) as per the number of hematomas. Pooled incidence of recurrences were 12.8% (95% CI 11.4-14.2; I^2^ = 86.1%; 95% PI 2.0-25.0) and 12.0% (95% CI 9.4-14.7; I^2^ = 0.0%), respectively (see **Supplementary Fig. 3** for forest plot, http://links.lww.com/ONS/A925). The pooled incidence of patients on antithrombotic medication, history of trauma, and history of alcohol abuse is 28.2% (95% CI 23.4-33.1; I^2^ = 98.2%), 64.4% (95% CI 55.5-73.3; I^2^ = 99%), and 15.3% (95% CI 10.8-19.8; I^2^ = 97.2%), respectively. Recurrence rates in high-quality studies per the irrigation method were as follows: saline/normal saline/isotonic saline/physiological saline, 13.8%, Ringer solution/Hartmann solution, 9.6%; gentamicin-induced irrigation method, 9.6% (determined in 1 study); warm saline, 10.7%; and warm Ringer solution of 10.0% (determined in 1 study). For the type of drainage method in high-quality studies, recurrence rate for a subdural drain was 13.6% and for subperiosteal drain was 15.8% (determined from 1 study), and a subgaleal drain was not used in high-quality studies. Regarding the duration of postoperative drainage times ≤24 hours, ≤48 hours, ≤72 hours, ≤96 hours, and ≤120 hours, the respective recurrence rates are 14.1%, 12.6%, 13.0%, 13.4% (determined in 1 study), and 13.6%.

### Definitions of Recurrence Used

In 167 articles, a definition of a recurrent cSDH was stated. In 66 of these articles, reoperation was the sole definition of recurrence. In 57 articles, a combination of clinical, radiological factors and a reoperation was regarded as recurrence; in 28 articles, clinical and radiological factors defined recurrence. In 2 articles, solely clinical symptoms were regarded as a recurrence and in 14 articles only radiological features. We determined the recurrence rates per definition, which are 10.5%, 12.4%, 13.9%, 14.0%, and 9.4%, respectively. In 29 articles, a period of 3 months was used for the recurrence definition and in 24 articles a period of 6 months. In the subgroup of 50 studies with the highest quality, 23 articles used a combination of clinical, radiological factors and a reoperation as definition of recurrence. In 9 articles clinical and radiological factors determined a recurrence and in 4 articles only radiological factors. Furthermore, 17 of these high-quality studies used a period of 3 months and 7 used a period of 6 months. We analyzed the pooled incidences and recurrence rates of treatment modalities of the studies using a combination of clinical, radiological factors and a reoperation as recurrence definition (**Supplementary Tables 2-4**, http://links.lww.com/ONS/A926, http://links.lww.com/ONS/A927, and http://links.lww.com/ONS/A928).

## DISCUSSION

This systematic review shows a pooled incidence of recurrence of 12.8% after burr-hole surgery and postoperative drainage in patients with a chronic subdural hematoma. Because this recurrence rate has been accurately determined, it can serve as an outcome measure for power calculations to determine sample sizes for future randomized clinical trials.

Most of the included studies were of a retrospective nature, the majority was judged to carry a high to very high risk of bias, and the RCTs were all considered to be of low quality. The overall pooled incidence is 11.2%. In our subgroup analysis of only those studies of the highest quality, the pooled incidence of recurrence was remarkably higher at 12.8%, which could infer that studies with poor quality systematically underestimate the recurrence rate. Therefore, the most reliable pooled incidence of recurrence is 12.8%. Our analyses of the pooled incidence of recurrence at 3 and 6 months shows that both periods do not accurately approach the overall recurrence rate of cSDH, possibly because of the low number of studies of which data could be derived from.

Recurrence of a cSDH leads to rehospitalization and reoperation, resulting in worse clinical outcome, loss of independency in these often very frail patients, and a significant increase in health care costs.^205-208^ Prevention of recurrences is therefore important, and various nonsurgical treatments are now being explored in randomized clinical trials, including medication (tranexamic acid).^9,209^ In a recent RCT, the use of dexamethasone vs placebo demonstrated that fewer recurrences occurred but that less favorable outcomes and more adverse events were noted in the dexamethasone group.^10^

This systematic review also provided information on recurrence rates depending on the method of irrigation used. Ringer solution (also titled Hartmann solution) showed a relatively high recurrence rate, as did irrigation fluid at room temperature (22 °C) when compared with irrigation at body temperature (37 °C), which is in line with a recent study.^210^ The pooled recurrence rate of subgaleal drainage was lower compared with subdural and subperiosteal drainage, although subgaleal drainage was used in only 8 studies, making it hard to draw firm conclusions. In the literature, recurrence rates after burr-hole surgery do not appear to be affected by drain location.^211^ However, 2 recent systematic reviews and meta-analysis showed that the insertion of a subperiosteal drain resulted in a significantly lower recurrence rate compared with a subdural drain but did not take into account a subgaleal drain.^212,213^ Furthermore, we researched whether patient characteristics or treatment modalities could be of influence on the high-quality recurrence rate by performing subgroup analyses. Only the irrigation method, saline/normal saline/isotonic saline/physiological saline, seems to be associated with recurrence because this rate is higher compared with the other determined rates in between treatment modalities. The pooled incidences of recurrence in the high-quality group could therefore be regarded as more stable and should be used as most reliable recurrence rates.

### Limitations

A relatively high statistical heterogeneity was observed for nearly all calculated pooled incidences in the current systematic review and meta-analysis. This could be due to clinical heterogeneity because clinical diversity in studies, despite the strict inclusion and exclusion criteria, is inevitable. A difference in baseline characteristics of included studies was observed. Some studies for example applied certain age restrictions, and some only included unilateral hematomas or patients with antithrombotic therapy, and across studies, different treatment methods regarding number of burr-holes, type of irrigation, and location of the postoperative drain and different definitions of recurrence were used. Moreover, methodological heterogeneity also attributes to the statistical heterogeneity because retrospective and prospective studies and RCTs are included. When measuring prevalence of a phenomenon in diverse environments, it is expected that highly heterogeneous studies are assembled.^214^ Because all included studies present a recurrence rate and therefore essentially measure the same parameter, we think it is worth summarizing despite clinical and methodological heterogeneity. Furthermore, in the literature, a wide range of recurrence rates are used, and the current study is a first step to accurately provide one overall recurrence rate. A subgroup analysis of studies with the highest quality was performed and still showed high heterogeneity meaning that even in these studies clinical and methodological differences are present and cannot be avoided.

Prediction interval calculation of studies with the highest quality showed that the recurrence rate in 95% of future studies is expected to range from 2% to 25%. This wide prediction interval is likely caused by the high heterogeneity of studies because the prediction interval is a summary of the spread of underlying effects in the studies included in the random-effects meta-analysis.^16^ The created forest plots show a considerable in-between study difference regarding recurrence rate, even in the studies with the highest quality. Consequently, the calculated pooled recurrence rate should be interpreted with some caution.

Fortunately, randomized controlled trials are being increasingly conducted in neurosurgery. However, for power calculations to determine sample size, it is crucial to use accurate outcome measures. The current study provides the most accurate known recurrence rate for cSDH treated by burr-hole surgery and postoperative drainage. Therefore, we propose to use the recurrence rate calculated in this study for future sample size calculations. Furthermore, to compare the results of these clinical studies, consistency in terms and definitions is essential, whereas definitions for a recurrent cSDH vary considerably in the literature. We would like to recommend using the following standardized definition of a recurrent cSDH in future studies: A recurrent cSDH is defined as recurring or aggravated clinical symptoms, caused by a radiologically proven, ipsilateral reaccumulation of cSDH within 6 months of prior surgical treatment, for which additional treatment is necessary. It then still remains debatable whether a recurrence on the contralateral side is a recurrence or a newly diagnosed cSDH.

## CONCLUSION

The overall recurrence rate of chronic subdural hematoma after burr-hole surgery and postoperative drainage is 12.8%. This rate should be interpreted with some caution because observed heterogeneity of included studies is high. A unified definition of cSDH recurrence after surgical treatment is advocated to ensure reliable comparison between future studies.

## Supplementary Material

## SUPPLEMENTAL DIGITAL CONTENT

**Supplemental Content Table 1**. Syntax search. **Supplementary Fig 1**. Forest plot and prediction interval of pooled incidence of recurrences per number of patients. **Supplementary Fig 2**. Forest plot and prediction interval of pooled incidence of recurrences per number of hematomas. **Supplementary Fig 3**. Forest plot and prediction interval of pooled incidence of recurrences of studies with the highest quality. **Supplemental Content Table 2**. Pooled incidence and recurrence rate of irrigation methods in studies using a definition of clinical and radiological factors and a reoperation. **Supplemental Content Table 3**. Pooled incidence and recurrence rate of postoperative drain location used in studies using a definition of clinical and radiological factors and a reoperation. **Supplemental Content Table 4**. Pooled incidence and recurrence rate of postoperative drainage time used in studies using a definition of clinical and radiological factors and a reoperation.

## COMMENTS

This systematic review and meta-analysis provide valuable insights into the recurrence rates of chronic subdural hematoma (cSDH) after burr-hole evacuation with postoperative drainage. While several reviews and meta-analyses have been published on this topic, this study stands out for its focus on recurrence rates and its unique analysis of the impact of irrigation solutions.

The review's findings suggest that the recurrence rate of cSDH after burr-hole evacuation with postoperative drainage is 12.8%, providing neurosurgeons with important information to inform their decision-making processes. Moreover, the study's examination of the effects of different irrigation solutions on recurrence rates provides additional insight into potential interventions that could improve patient outcomes.

Overall, this review underscores the importance of ongoing research into the management of cSDH and the need for clinicians to carefully consider the specific factors that may impact recurrence rates when making treatment decisions. The study's rigorous methodology and clear reporting of results make it a valuable contribution to the current literature on this topic.

**Jason H. Huang**

*Temple, Texas, USA*
