# Supplementary material for: Recurrent Chronic Subdural Hematoma After Burr-Hole Surgery and Postoperative Drainage: A Systematic Review and Meta-Analysis
Source: Oper Neurosurg. 2023 Jun 30;25(3):216–41. doi: 10.1227/ons.0000000000000794 (PMC10389757; doi:10.1227/ons.0000000000000794)

Author	g	SE	Proportion	95%−CI	Weight (fixed)	Weight (random)
Aung 1999 [1]	0	50	0.00	[0.00; 0.07]	1.2%	0.7%
Jukovic 2014 [2]	0	35	0.00	[0.00; 0.10]	0.6%	0.7%
Kaliaperumal 2012 [3]	0	50	0.00	[0.00; 0.07]	1.2%	0.7%
Wang 2017 [4]	0	57	0.00	[0.00; 0.06]	1.6%	0.7%
Yagnick 2019 [5]	0	60	0.00	[0.00; 0.06]	1.7%	0.7%
Thavara 2019 [6]	1	63	0.02	[0.00; 0.09]	0.9%	0.7%
Chandran 2017 [7]	1	52	0.02	[0.00; 0.10]	0.6%	0.7%
Singh 2011 [8]	1	52	0.02	[0.00; 0.10]	0.6%	0.7%
Choudhury 1994 [9]	1	44	0.02	[0.00; 0.12]	0.5%	0.6%
Kotwica 1991 [10]	3	131	0.02	[0.00; 0.07]	1.4%	0.7%
Liu 2019 [11]	8	328	0.02	[0.01; 0.05]	3.2%	0.7%
Kutty 2014 [12]	2	70	0.03	[0.00; 0.10]	0.6%	0.7%
Ishibashi 2011 [13]	1	34	0.03	[0.00; 0.15]	0.3%	0.6%
Suzuki 1998 [14]	2	67	0.03	[0.00; 0.10]	0.5%	0.7%
Djientcheu 2011 [15]	6	195	0.03	[0.01; 0.07]	1.5%	0.7%
Markwalder 2000 [16]	1	32	0.03	[0.00; 0.16]	0.2%	0.6%
Certo 2019 [17]	1	30	0.03	[0.00; 0.17]	0.2%	0.5%
Sucu 2014 [18]	4	119	0.03	[0.01; 0.08]	0.9%	0.7%
Vasella 2018 [19]	1	28	0.04	[0.00; 0.18]	0.2%	0.5%
Yadav 2016 [20]	5	140	0.04	[0.01; 0.08]	0.9%	0.7%
Liu 2010 [21]	15	398	0.04	[0.02; 0.06]	2.6%	0.7%
Liliang 2002 [22]	3	75	0.04	[0.01; 0.11]	0.5%	0.6%
Kwon 2000 [23]	6	145	0.04	[0.02; 0.09]	0.9%	0.7%
Penchet 1998 [24]	10	236	0.04	[0.02; 0.08]	1.4%	0.7%
Lu 2018 [25]	4	87	0.05	[0.01; 0.11]	0.5%	0.6%
Adrian 2017 [26]	3	60	0.05	[0.01; 0.14]	0.3%	0.6%
Kanyi 2018 [27]	6	119	0.05	[0.02; 0.11]	0.6%	0.7%
Oral 2015 [28]	4	78	0.05	[0.01; 0.13]	0.4%	0.6%
Lee 2018 [29]	7	131	0.05	[0.02; 0.11]	0.6%	0.7%
Sousa 2013 [30]	42	778	0.05	[0.04; 0.07]	3.6%	0.7%
Lepic 2021 [31]	3	55	0.05	[0.01; 0.15]	0.2%	0.6%
Han 2009 [32]	10	180	0.06	[0.03; 0.10]	0.8%	0.7%
Tommiska 2019 [33]	4	71	0.06	[0.02; 0.14]	0.3%	0.6%
Flores 2017 [34]	13	220	0.06	[0.03; 0.10]	0.9%	0.7%
Gelabert Gonzalez 2005 [35]	61	1000	0.06	[0.05; 0.08]	4.1%	0.7%
Jeong 2014 [36]	8	125	0.06	[0.03; 0.12]	0.5%	0.6%
Missori 2000 [37]	2	31	0.06	[0.01; 0.21]	0.1%	0.5%
Leung 2001 [38]	3	46	0.07	[0.01; 0.18]	0.2%	0.5%
Regan 2015 [39]	4	61	0.07	[0.02; 0.16]	0.2%	0.6%
Mersha 2020 [40]	13	195	0.07	[0.04; 0.11]	0.7%	0.7%
Wang 2017 [42]	6	88	0.07	[0.03; 0.14]	0.3%	0.6%
Kiymaz 2007 [42]	2	29	0.07	[0.01; 0.23]	0.1%	0.4%
Choi 2016 [43]	37	502	0.07	[0.05; 0.10]	1.7%	0.7%
Kurabe 2010 [44]	14	182	0.08	[0.04; 0.13]	0.6%	0.7%
Mezue 2011 [45]	9	116	0.08	[0.04; 0.14]	0.4%	0.6%
Glancz 2019 [46]	45	577	0.08	[0.06; 0.10]	1.9%	0.7%
Tomita 2018 [47]	8	102	0.08	[0.03; 0.15]	0.3%	0.6%
Kang 2007 [48]	24	302	0.08	[0.05; 0.12]	1.0%	0.7%
Piotrowski 1996 [49]	16	200	0.08	[0.05; 0.13]	0.6%	0.7%
Dran 2007 [50]	16	198	0.08	[0.05; 0.13]	0.6%	0.7%
Tailor 2017 [51]	10	123	0.08	[0.04; 0.14]	0.4%	0.6%
Yu 2009 [52]	8	97	0.08	[0.04; 0.16]	0.3%	0.6%
Carlisi 2017 [53]	3	35	0.09	[0.02; 0.23]	0.1%	0.4%
Huang 2020 [54]	12	140	0.09	[0.05; 0.14]	0.4%	0.6%
Bartley 2020 [55]	15	172	0.09	[0.05; 0.14]	0.5%	0.6%
Chan 2017 [56]	13	149	0.09	[0.05; 0.14]	0.4%	0.6%
Sah 2018 [57]	9	102	0.09	[0.04; 0.16]	0.3%	0.6%
Kim 2011 [58]	23	259	0.09	[0.06; 0.13]	0.7%	0.7%
Okano 2014 [59]	40	448	0.09	[0.06; 0.12]	1.3%	0.7%
Goto 2015 [60]	37	414	0.09	[0.06; 0.12]	1.2%	0.7%
Weng 2019 [61]	17	190	0.09	[0.05; 0.14]	0.5%	0.7%
Singh 2014 [62]	9	100	0.09	[0.04; 0.16]	0.3%	0.6%
Wu 2020 [63]	30	331	0.09	[0.06; 0.13]	0.9%	0.7%
Flint 2017 [64]	60	659	0.09	[0.07; 0.12]	1.9%	0.7%
Fujisawa 2021 [65]	19	208	0.09	[0.06; 0.14]	0.6%	0.7%
Adachi 2014 [66]	11	120	0.09	[0.05; 0.16]	0.3%	0.6%
Santarius 2009 [67]	10	108	0.09	[0.05; 0.16]	0.3%	0.6%
Tsai 2010 [68]	12	129	0.09	[0.05; 0.16]	0.4%	0.6%
Hsieh 2016 [69]	7	75	0.09	[0.04; 0.18]	0.2%	0.5%
Li 2017 [70]	11	115	0.10	[0.05; 0.16]	0.3%	0.6%
Mori 2001 [71]	49	500	0.10	[0.07; 0.13]	1.3%	0.7%
Jang 2020 [72]	29	291	0.10	[0.07; 0.14]	0.8%	0.7%
Jang 2015 [73]	3	30	0.10	[0.02; 0.27]	0.1%	0.4%
Zakaraia 2008 [74]	4	40	0.10	[0.03; 0.24]	0.1%	0.4%
Hamilton 1993 [75]	3	29	0.10	[0.02; 0.27]	0.1%	0.4%
Yamamoto 2003 [76]	11	105	0.10	[0.05; 0.18]	0.3%	0.6%
Abouzari 2007 [77]	9	84	0.11	[0.05; 0.19]	0.2%	0.5%
Eggert 1984 [78]	11	100	0.11	[0.06; 0.19]	0.2%	0.6%
Yamada 2018 [79]	119	1080	0.11	[0.09; 0.13]	2.6%	0.7%
Kareem 2018 [80]	4	36	0.11	[0.03; 0.26]	0.1%	0.4%
Kuroki 2001 [81]	5	45	0.11	[0.04; 0.24]	0.1%	0.4%
Wang 2017 [82]	5	45	0.11	[0.04; 0.24]	0.1%	0.4%
Ak 2017 [83]	8	71	0.11	[0.05; 0.21]	0.2%	0.5%
Ishfaq 2017 [84]	7	62	0.11	[0.05; 0.22]	0.1%	0.5%
Toi 2019 [85]	39	342	0.11	[0.08; 0.15]	0.8%	0.7%
Chang 2020 [86]	14	122	0.11	[0.06; 0.19]	0.3%	0.6%
Blaauw 2020 [87]	115	995	0.12	[0.10; 0.14]	2.3%	0.7%
Morales−Gomez 2020 [88]	18	155	0.12	[0.07; 0.18]	0.4%	0.6%
Ryu 2018 [89]	22	187	0.12	[0.08; 0.17]	0.4%	0.6%
Lin 2011 [90]	32	270	0.12	[0.08; 0.16]	0.6%	0.7%
Rovlias 2015 [91]	117	986	0.12	[0.10; 0.14]	2.2%	0.7%
Cheng 2014 [92]	41	342	0.12	[0.09; 0.16]	0.8%	0.7%
Shah 2014 [93]	3	25	0.12	[0.03; 0.31]	0.1%	0.3%
Gabarros 2000 [94]	10	83	0.12	[0.06; 0.21]	0.2%	0.5%
Tanikawa 2001 [95]	4	33	0.12	[0.03; 0.28]	0.1%	0.4%
Motoie 2018 [96]	96	787	0.12	[0.10; 0.15]	1.7%	0.7%
Zhang 2019 [97]	70	570	0.12	[0.10; 0.15]	1.2%	0.7%
Bankole 2011 [98]	9	73	0.12	[0.06; 0.22]	0.2%	0.5%
ShafiqAlam 2017 [99]	53	428	0.12	[0.09; 0.16]	0.9%	0.7%
Katayama 2018 [100]	11	88	0.12	[0.06; 0.21]	0.2%	0.5%
Kim 2017 [101]	31	246	0.13	[0.09; 0.17]	0.5%	0.7%
Castro Rodriguez 2016 [102]	26	200	0.13	[0.09; 0.18]	0.4%	0.6%
Tagle 2003 [103]	13	100	0.13	[0.07; 0.21]	0.2%	0.5%
Nunta−Aree 2017 [104]	10	75	0.13	[0.07; 0.23]	0.2%	0.5%
Shim 2019 [105]	8	60	0.13	[0.06; 0.25]	0.1%	0.5%
Sjavik 2017 [106]	169	1260	0.13	[0.12; 0.15]	2.5%	0.7%
Yan 2017 [107]	7	52	0.13	[0.06; 0.26]	0.1%	0.4%
Bartek 2017 [108]	169	1254	0.13	[0.12; 0.15]	2.5%	0.7%
Ram 1993 [109]	5	37	0.14	[0.05; 0.29]	0.1%	0.4%
Baechli 2004 [110]	48	354	0.14	[0.10; 0.18]	0.7%	0.7%
Jung 2015 [111]	25	182	0.14	[0.09; 0.20]	0.4%	0.6%
Dobran 2019 [112]	7	50	0.14	[0.06; 0.27]	0.1%	0.4%
Carlsen 2011 [113]	29	206	0.14	[0.10; 0.20]	0.4%	0.6%
Erol 2005 [114]	5	35	0.14	[0.05; 0.30]	0.1%	0.3%
Frati 2004 [115]	5	35	0.14	[0.05; 0.30]	0.1%	0.3%
Huang 2013 [116]	14	98	0.14	[0.08; 0.23]	0.2%	0.5%
Lo 2013 [117]	14	98	0.14	[0.08; 0.23]	0.2%	0.5%
Yan 2018 [118]	33	231	0.14	[0.10; 0.19]	0.4%	0.6%
Gonugunta 2001 [119]	27	184	0.15	[0.10; 0.21]	0.3%	0.6%
Wang 2020 [120]	96	653	0.15	[0.12; 0.18]	1.2%	0.7%
Tugcu 2014 [121]	43	292	0.15	[0.11; 0.19]	0.5%	0.7%
D'Oria 2020 [122]	31	210	0.15	[0.10; 0.20]	0.4%	0.6%
Zumofen 2009 [123]	22	147	0.15	[0.10; 0.22]	0.3%	0.6%
You 2018 [124]	34	226	0.15	[0.11; 0.20]	0.4%	0.6%
Shen 2019 [125]	69	457	0.15	[0.12; 0.19]	0.8%	0.7%
Sarnvivad 2011 [126]	15	97	0.15	[0.09; 0.24]	0.2%	0.5%
Janowski 2012 [127]	7	45	0.16	[0.06; 0.29]	0.1%	0.4%
Heringer 2017 [128]	15	96	0.16	[0.09; 0.24]	0.2%	0.5%
Raghavan 2020 [129]	24	153	0.16	[0.10; 0.22]	0.3%	0.6%
Lee 2004 [130]	6	38	0.16	[0.06; 0.31]	0.1%	0.3%
Maldaner 2019 [131]	40	253	0.16	[0.12; 0.21]	0.4%	0.6%
Poulsen 2014 [132]	28	177	0.16	[0.11; 0.22]	0.3%	0.6%
Stanisic 2013 [133]	17	107	0.16	[0.10; 0.24]	0.2%	0.5%
Bellut 2012 [134]	18	113	0.16	[0.10; 0.24]	0.2%	0.5%
Ahmed 2011 [135]	4	25	0.16	[0.05; 0.36]	0.0%	0.3%
Eppel 1999 [136]	8	50	0.16	[0.07; 0.29]	0.1%	0.4%
Qian 2017 [137]	39	242	0.16	[0.12; 0.21]	0.4%	0.6%
Hori 2018 [138]	15	92	0.16	[0.09; 0.25]	0.2%	0.5%
Song 2014 [139]	16	97	0.16	[0.10; 0.25]	0.2%	0.5%
Kocaman 2019 [140]	5	30	0.17	[0.06; 0.35]	0.1%	0.3%
Lee 2014 [141]	19	114	0.17	[0.10; 0.25]	0.2%	0.5%
Wang 2016 [142]	9	53	0.17	[0.08; 0.30]	0.1%	0.4%
Gilsbach 1980 [143]	9	51	0.18	[0.08; 0.31]	0.1%	0.4%
Iftikhar 2016 [144]	6	34	0.18	[0.07; 0.35]	0.1%	0.3%
Klein 2021 [145]	72	407	0.18	[0.14; 0.22]	0.7%	0.7%
Miki 2019 [146]	50	277	0.18	[0.14; 0.23]	0.4%	0.6%
Ernestus 1997 [147]	17	94	0.18	[0.11; 0.27]	0.1%	0.5%
Torihashi 2008 [148]	61	337	0.18	[0.14; 0.23]	0.5%	0.7%
Schwarz 2015 [149]	35	193	0.18	[0.13; 0.24]	0.3%	0.6%
Nakagawa 2019 [150]	71	381	0.19	[0.15; 0.23]	0.6%	0.7%
Ridwan 2019 [151]	37	197	0.19	[0.14; 0.25]	0.3%	0.6%
Drapkin 1991 [152]	10	53	0.19	[0.09; 0.32]	0.1%	0.4%
Martinez Perez 2020 [153]	17	90	0.19	[0.11; 0.29]	0.1%	0.5%
Abboud 2018 [154]	38	201	0.19	[0.14; 0.25]	0.3%	0.6%
Gurelik 2007 [155]	8	42	0.19	[0.09; 0.34]	0.1%	0.3%
Choi 2020 [156]	49	230	0.21	[0.16; 0.27]	0.3%	0.6%
Edem 2019 [157]	16	74	0.22	[0.13; 0.33]	0.1%	0.4%
Miah 2020 [158]	13	60	0.22	[0.12; 0.34]	0.1%	0.4%
Lee 2009 [159]	7	32	0.22	[0.09; 0.40]	0.0%	0.3%
Chon 2012 [160]	92	420	0.22	[0.18; 0.26]	0.6%	0.7%
Schoedel 2016 [161]	155	697	0.22	[0.19; 0.26]	0.9%	0.7%
Munoz−Bendix 2017 [162]	25	112	0.22	[0.15; 0.31]	0.2%	0.5%
Kristof 2008 [163]	17	75	0.23	[0.14; 0.34]	0.1%	0.4%
Won 2021 [164]	40	176	0.23	[0.17; 0.30]	0.2%	0.6%
Hani 2019 [165]	83	361	0.23	[0.19; 0.28]	0.5%	0.6%
Khan 2019 [166]	14	60	0.23	[0.13; 0.36]	0.1%	0.4%
Harders 1982 [167]	24	100	0.24	[0.16; 0.34]	0.1%	0.5%
Kim 2014 [168]	28	114	0.25	[0.17; 0.34]	0.1%	0.5%
Krupp 1995 [169]	53	214	0.25	[0.19; 0.31]	0.3%	0.6%
Tahsim−Oglou 2012 [170]	62	247	0.25	[0.20; 0.31]	0.3%	0.6%
Zhang 2018 [171]	8	31	0.26	[0.12; 0.45]	0.0%	0.2%
Kim 2016 [172]	26	100	0.26	[0.18; 0.36]	0.1%	0.5%
Rohde 2002 [173]	119	376	0.32	[0.27; 0.37]	0.4%	0.6%
Wan 2017 [174]	13	31	0.42	[0.25; 0.61]	0.0%	0.2%
**Fixed effect model**		**34393**	**0.09**	**[0.09; 0.09]**	**100.0%**	**−−**
**Random effects model**			**0.11**	**[0.10; 0.12]**	**−−**	**100.0%**
**Prediction interval**				**[0.00; 0.22]**		

Heterogeneity: $I^2 = 88\%$, $p < 0.01$

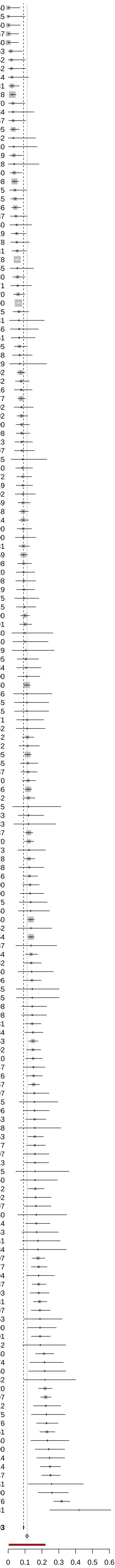

Supplement: Supplementary file 1 [file ons-25-216-s001.pdf]
