# Supplementary figures and images for: Recurrent Chronic Subdural Hematoma After Burr-Hole Surgery and Postoperative Drainage: A Systematic Review and Meta-Analysis
Source: Oper Neurosurg. 2023 Jun 30;25(3):216–41. doi: 10.1227/ons.0000000000000794 (PMC10389757; doi:10.1227/ons.0000000000000794)

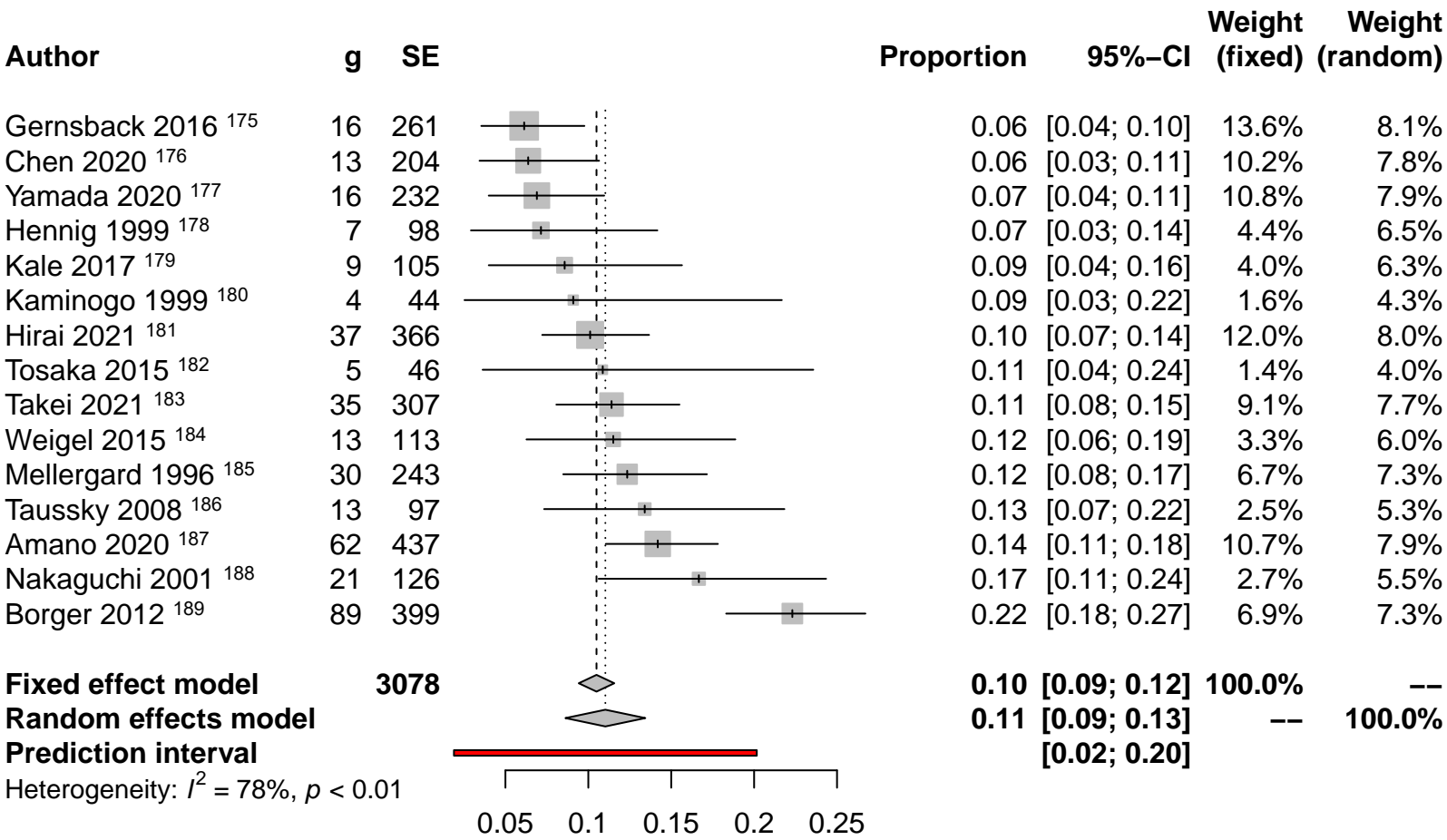

Supplement: Supplementary file 2 [file ons-25-216-s002.pdf]
