# Supplementary material for: Recurrent Chronic Subdural Hematoma After Burr-Hole Surgery and Postoperative Drainage: A Systematic Review and Meta-Analysis
Source: Oper Neurosurg. 2023 Jun 30;25(3):216–41. doi: 10.1227/ons.0000000000000794 (PMC10389757; doi:10.1227/ons.0000000000000794)
Supplement: Supplementary file 3 [file ons-25-216-s003.pdf]

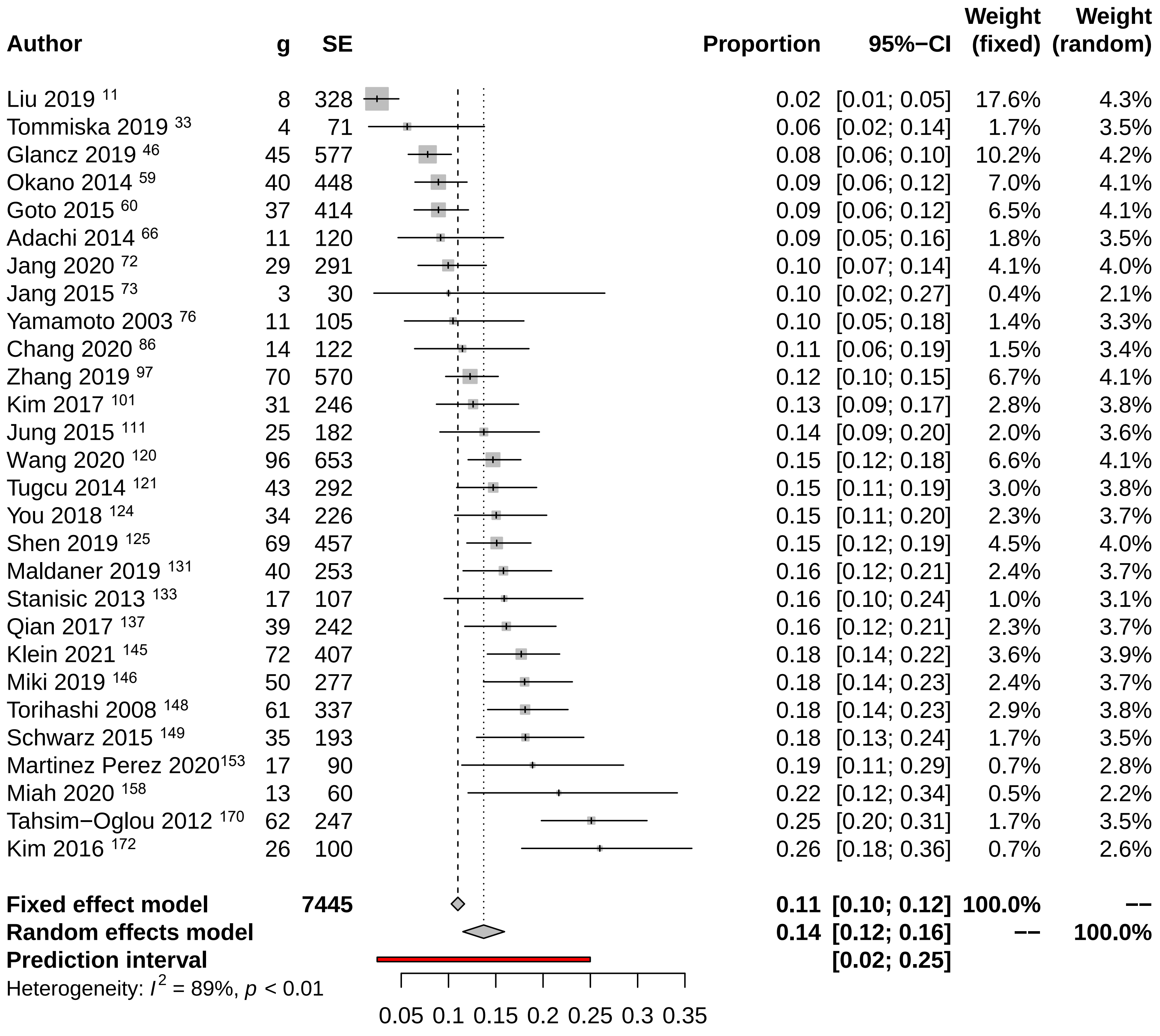
Author g SE Proportion 95%−CI Weight (fixed) Weight (random)
Liu 2019 11 8 328 0.02 [0.01; 0.05] 17.6% 4.3%
Tommiska 2019 33 4 71 0.06 [0.02; 0.14] 1.7% 3.5%
Glancz 2019 46 45 577 0.08 [0.06; 0.10] 10.2% 4.2%
Okano 2014 59 40 448 0.09 [0.06; 0.12] 7.0% 4.1%
Goto 2015 60 37 414 0.09 [0.06; 0.12] 6.5% 4.1%
Adachi 2014 66 11 120 0.09 [0.05; 0.16] 1.8% 3.5%
Jang 2020 72 29 291 0.10 [0.07; 0.14] 4.1% 4.0%
Jang 2015 73 3 30 0.10 [0.02; 0.27] 0.4% 2.1%
Yamamoto 2003 76 11 105 0.10 [0.05; 0.18] 1.4% 3.3%
Chang 2020 86 14 122 0.11 [0.06; 0.19] 1.5% 3.4%
Zhang 2019 97 70 570 0.12 [0.10; 0.15] 6.7% 4.1%
Kim 2017 101 31 246 0.13 [0.09; 0.17] 2.8% 3.8%
Jung 2015 111 25 182 0.14 [0.09; 0.20] 2.0% 3.6%
Wang 2020 120 96 653 0.15 [0.12; 0.18] 6.6% 4.1%
Tugcu 2014 121 43 292 0.15 [0.11; 0.19] 3.0% 3.8%
You 2018 124 34 226 0.15 [0.11; 0.20] 2.3% 3.7%
Shen 2019 125 69 457 0.15 [0.12; 0.19] 4.5% 4.0%
Maldaner 2019 131 40 253 0.16 [0.12; 0.21] 2.4% 3.7%
Stanisic 2013 133 17 107 0.16 [0.10; 0.24] 1.0% 3.1%
Qian 2017 137 39 242 0.16 [0.12; 0.21] 2.3% 3.7%
Klein 2021 145 72 407 0.18 [0.14; 0.22] 3.6% 3.9%
Miki 2019 146 50 277 0.18 [0.14; 0.23] 2.4% 3.7%
Torihashi 2008 148 61 337 0.18 [0.14; 0.23] 2.9% 3.8%
Schwarz 2015 149 35 193 0.18 [0.13; 0.24] 1.7% 3.5%
Martinez Perez 2020153 17 90 0.19 [0.11; 0.29] 0.7% 2.8%
Miah 2020 158 13 60 0.22 [0.12; 0.34] 0.5% 2.2%
Tahsim−Oglou 2012 170 62 247 0.25 [0.20; 0.31] 1.7% 3.5%
Kim 2016 172 26 100 0.26 [0.18; 0.36] 0.7% 2.6%
Fixed effect model 7445 0.11 [0.10; 0.12] 100.0% −−
Random effects model 0.14 [0.12; 0.16] −− 100.0%
Prediction interval [0.02; 0.25]
Heterogeneity: I2 = 89%, p < 0.01
0.05 0.1 0.15 0.2 0.25 0.3 0.35

References Figures 1-3

1. Aung TH, Wong WK, Mo HP, Tsang CS. Management of chronic subdural haematoma: burr hole drainage, replacement with Hartmann's solution, and closed-system drainage. *Hong Kong Med J.* 1999;5(4):383-386.
2. Jukovic M, Till V, Backalic T, Karan M, Petric G. The use of the Karnofsky index in the assessment of clinical state in patients with chronic subdural hematoma: the first observation from Vojvodina. *Medicinski Glasnik Ljekarske Komore Zenickodobojskog Kantona.* 2014;11(1):132-137.
3. Kaliaperumal C, Khalil A, Fenton E, et al. A prospective randomised study to compare the utility and outcomes of subdural and subperiosteal drains for the treatment of chronic subdural haematoma. *Acta Neurochir (Wien).* 2012;154(11):2083-2088; discussion 2088-2089.
4. Wang W, Liu H, Yang J. Burr hole craniostomy irrigation with and without drainage during surgical treatment of chronic subdural hematoma: A retrospective study of 87 cases. *Turkish Neurosurgery.* 2017;31:31.
5. Yagnick NS, Moolchandani S, Sinha S, Mehta VS. Demonstration of Brain Expansion in Cases of Chronic SDH during Admission Leads to Decreased Rates of Recurrence. *Indian Journal of Neurotrauma.* 2019;16(1):63-66.
6. Thavara BD, Kidangan GS, Rajagopalawarrier B. Comparative Study of Single Burr-Hole Craniostomy versus Twist-Drill Craniostomy in Patients with Chronic Subdural Hematoma. *Asian Journal of Neurosurgery.* 2019;14(2):513-521.
7. Chandran RS, Nagar M, Sharmad MS, et al. Single Parietal Burr-hole Craniostomy with Irrigation and Drainage for Unilateral Chronic Subdural Hematoma in Young Adults <40 Years: A Rationale behind the Procedure. *J Neurosci Rural Pract.* 2017;8(3):389-394.
8. Singh S.K. SM, Singh V.K., Parihar A., Srivastava C., Ojha B.K., Chandra A. A randomized study of twist drill versus burr hole craniostomy for treatment of chronic subdural hematomas in 100 patients. *The Indian Journal of Neurotrauma.* 2011;8(2):83-88.
9. Choudhury AR. Avoidable factors that contribute to complications in the surgical treatment of chronic subdural haematoma. *Acta Neurochir (Wien).* 1994;129(1-2):15-19.
10. Kotwica Z, Brzezinski J. Chronic subdural haematoma treated by burr holes and closed system drainage: personal experience in 131 patients. *Br J Neurosurg.* 1991;5(5):461-465.
11. Liu LX, Cao XD, Ren YM, Zhou LX, Yang CH. Risk Factors for Recurrence of Chronic Subdural Hematoma: A Single Center Experience. *World Neurosurgery.* 2019;132:e506-e513.
12. Kutty SA, Johny M. Chronic subdural hematoma: a comparison of recurrence rates following burr-hole craniostomy with and without drains. *Turk Neurosurg.* 2014;24(4):494-497.
13. Ishibashi A, Yokokura Y, Adachi H. A comparative study of treatments for chronic subdural hematoma: burr hole drainage versus burr hole drainage with irrigation. *Kurume Med J.* 2011;58(1):35-39.
14. Suzuki K, Sugita K, Akai T, Takahata T, Sonobe M, Takahashi S. Treatment of chronic subdural hematoma by closed-system drainage without irrigation. *Surgical Neurology.* 1998;50(3):231-234.
15. Djientcheu VP, Esiene A, Yamgoue T, Tchaleu B, Ze Minkande J. Surgical treatment and outcome of 195 cases of non acute subdural haematoma at the Yaoundé Central Hospital: The need for landmarked burr holes. *African Journal of Neurological Sciences.* 2011;30(2).
16. Markwalder TM. The course of chronic subdural hematomas after burr-hole craniostomy with and without closed-system drainage. *Neurosurgery Clinics of North America.* 2000;11(3):541-546.

17. Certo F, Maione M, Altieri R, et al. Pros and cons of a minimally invasive percutaneous subdural drainage system for evacuation of chronic subdural hematoma under local anesthesia. *Clin Neurol Neurosurg.* 2019;187:105559.
18. Sucu HK, Akar O. Double-layer appearance after evacuation of a chronic subdural hematoma. *British Journal of Neurosurgery.* 2014;28(1):93-97.
19. Vasella F, Akeret K, Smoll NR, et al. Improving the aesthetic outcome with burr hole cover placement in chronic subdural hematoma evacuation—a retrospective pilot study. *Acta Neurochirurgica.* 2018;160(11):2129-2135.
20. Yadav YR, Parihar V, Chourasia ID, Bajaj J, Namdev H. The role of subgaleal suction drain placement in chronic subdural hematoma evacuation. *Asian Journal of Neurosurgery.* 2016;11(3):214-218.
21. Liu Y, Xia JZ, Wu AH, Wang YJ. Burr-hole craniotomy treating chronic subdural hematoma: a report of 398 cases. *Chinese Journal of Traumatology.* 2010;13(5):265-269.
22. Liliang PC, Tsai YD, Liang CL, Lee TC, Chen HJ. Chronic subdural haematoma in young and extremely aged adults: A comparative study of two age groups. *Injury.* 2002;33(4):345-348.
23. Kwon TH, Park YK, Lim DJ, et al. Chronic subdural hematoma: evaluation of the clinical significance of postoperative drainage volume. *J Neurosurg.* 2000;93(5):796-799.
24. Penchet G, Loiseau H, Castel JP. [Chronic bilateral subdural hematomas]. *Neurochirurgie.* 1998;44(4):247-252.
25. Lu W, Wang H, Wu T, Sheng X, Ding Z, Xu G. Burr-Hole Craniostomy with T-Tube Drainage as Surgical Treatment for Chronic Subdural Hematoma. *World Neurosurgery.* 2018;115:e756-e760.
26. Adrian Ng WC, Albert Wong SH, Noor Azman AR, Jafri Malin A. Subperiosteal drainage versus subdural drainage in the management of chronic subdural hematoma (a comparative study). *Malaysian Journal of Medical Sciences.* 2017;24(1):21-30.
27. Kanyi JK, Ogada TV, Oloo MJ, Parker RK. Burr-Hole Craniostomy for Chronic Subdural Hematomas by General Surgeons in Rural Kenya. *World J Surg.* 2018;42(1):40-45.
28. Oral S, Borklu RE, Kucuk A, Ulutabanca H, Selcuklu A. Comparison of subgaleal and subdural closed drainage system in the surgical treatment of chronic subdural hematoma. *Northern Clinics of Istanbul.* 2015;2(2):115-121.
29. Lee SH, Choi JI, Lim DJ, Ha SK, Kim SD, Kim SH. The Potential of Diffusion-Weighted Magnetic Resonance Imaging for Predicting the Outcomes of Chronic Subdural Hematomas. *J Korean Neurosurg Soc.* 2018;61(1):97-104.
30. Sousa EB, Brandao LF, Tavares CB, Borges IB, Neto NG, Kessler IM. Epidemiological characteristics of 778 patients who underwent surgical drainage of chronic subdural hematomas in Brasilia, Brazil. *BMC Surgery.* 2013;13:5.
31. Lepic M, Mandic-Rajcevic S, Pavlicevic G, Novakovic N, Rasulic L. Awake surgery in sitting position for chronic subdural hematoma. *Acta Neurochirurgica.* 2021;19:19.
32. Han HJ, Park CW, Kim EY, Yoo CJ, Kim YB, Kim WK. One vs. Two Burr Hole Craniostomy in Surgical Treatment of Chronic Subdural Hematoma. *J Korean Neurosurg Soc.* 2009;46(2):87-92.
33. Tommiska P, Lonnrot K, Raj R, Luostarinen T, Kivisaari R. Transition of a Clinical Practice to Use of Subdural Drains after Burr Hole Evacuation of Chronic Subdural Hematoma: The Helsinki Experience. *World Neurosurgery.* 2019;129:e614-e626.
34. Flores G, Vicenty JC, Pastrana EA. Post-operative seizures after burr hole evacuation of chronic subdural hematomas: is prophylactic anti-epileptic medication needed? *Acta Neurochir (Wien).* 2017;159(11):2033-2036.
35. Gelabert-Gonzalez M, Iglesias-Pais M, Garcia-Allut A, Martinez-Rumbo R. Chronic subdural haematoma: surgical treatment and outcome in 1000 cases. *Clin Neurol Neurosurg.* 2005;107(3):223-229.

36. Jeong SI, Kim SO, Won YS, Kwon YJ, Choi CS. Clinical Analysis of Risk Factors for Recurrence in Patients with Chronic Subdural Hematoma Undergoing Burr Hole Trephination. *Korean J Neurotrauma.* 2014;10(1):15-21.
37. Missori P, Maraglino C, Tarantino R, et al. Chronic subdural haematomas in patients aged under 50. *Clin Neurol Neurosurg.* 2000;102(4):199-202.
38. Leung GKK, Fan JKM, Tam MCH, Fan YW. Surgical complications of chronic subdural haematoma: A 5-year audit. *Annals of the College of Surgeons of Hong Kong.* 2001;5(3):99-103.
39. Regan JM, Worley E, Shelburne C, Pullarkat R, Watson JC. Burr hole washout versus craniotomy for chronic subdural hematoma: patient outcome and cost analysis. *PLoS ONE [Electronic Resource].* 2015;10(1):e0115085.
40. Mersha A, Abat S, Temesgen T, Nebyou A. Outcome of Chronic Subdural Hematoma Treated with Single Burr Hole Under Local Anesthesia. *Ethiopian Journal of Health Sciences.* 2020;30(1):101-106.
41. Wang QP, Yuan Y, Guan JW, Jiang XB. A comparative study of irrigation versus no irrigation during burr hole craniostomy to treat chronic subdural hematoma. *BMC Surgery.* 2017;17(1):99.
42. Kiymaz N, Yilmaz N, Mumcu C. Controversies in chronic subdural hematoma: continuous drainage versus one-time drainage. *Med Sci Monit.* 2007;13(5):CR240-243.
43. Choi JJ, Kim HS, Lee KC, Hur H, Jo YY. Prediction of in-hospital mortality and morbidity using high-sensitivity C-reactive protein after burr hole craniostomy. *J Anesth.* 2016;30(6):956-960.
44. Kurabe S, Ozawa T, Watanabe T, Aiba T. Efficacy and safety of postoperative early mobilization for chronic subdural hematoma in elderly patients. *Acta Neurochir (Wien).* 2010;152(7):1171-1174.
45. Mezue WC, Ohaebgulam SC, Chikani MC, Erechukwu AU. Changing trends in chronic subdural haematoma in Nigeria. *Afr J Med Med Sci.* 2011;40(4):373-376.
46. Glancz LJ, Poon MTC, Coulter IC, Hutchinson PJ, Kolias AG, Brennan PM. Does Drain Position and Duration Influence Outcomes in Patients Undergoing Burr-Hole Evacuation of Chronic Subdural Hematoma? Lessons from a UK Multicenter Prospective Cohort Study. *Clinical Neurosurgery.* 2019;85(4):486-493.
47. Tomita Y, Yamada SM, Yamada S, Matsuno A. Subdural Tension on the Brain in Patients with Chronic Subdural Hematoma Is Related to Hemiparesis but Not to Headache or Recurrence. *World Neurosurgery.* 2018;119:e518-e526.
48. Kang MS, Koh HS, Kwon HJ, Choi SW, Kim SH, Youm JY. Factors influencing recurrent chronic subdural hematoma after surgery. *Journal of Korean Neurosurgical Society.* 2007;41(1):11-15.
49. Piotrowski WP, Krombholz-Reindl MA. [Surgical outcome in chronic subdural hematoma]. *Unfallchirurgie.* 1996;22(3):110-116.
50. Dran G, Berthier F, Fontaine D, Rasenrarijao D, Paquis P. [Effectiveness of adjuvant corticosteroid therapy for chronic subdural hematoma: a retrospective study of 198 cases]. *Neurochirurgie.* 2007;53(6):477-482.
51. Tailor J, Fernando D, Sidhu Z, Foley R, Abeysinghe KD, Walsh DC. Clinical audit effectively bridges the evidence-practice gap in chronic subdural haematoma management. *Acta Neurochirurgica.* 2017;159(4):627-631.
52. Yu GJ, Han CZ, Zhang M, Zhuang HT, Jiang YG. Prolonged drainage reduces the recurrence of chronic subdural hematoma. *British Journal of Neurosurgery.* 2009;23(6):606-611.
53. Carlisi E, Feltroni L, Tinelli C, Verlotta M, Gaetani P, Dalla Toffola E. Postoperative rehabilitation for chronic subdural hematoma in the elderly. An observational study focusing on balance, ambulation and discharge destination. *Eur J Phys Rehabil Med.* 2017;53(1):91-97.
54. Huang GH, Li XC, Ren L, et al. Take it seriously or not: postoperative pneumocephalus in CSDH patients? *Br J Neurosurg.* 2020;34(3):284-289.

55. Bartley A, Jakola AS, Tisell M. The influence of irrigation fluid temperature on recurrence in the evacuation of chronic subdural hematoma. *Acta Neurochir (Wien).* 2020;162(3):485-488.
56. Chan DY, Woo PY, Mak CH, et al. Use of subdural drain for chronic subdural haematoma? A 4-year multi-centre observational study of 302 cases. *Journal of Clinical Neuroscience.* 2017;36:27-30.
57. Sah S, Rawal D. Craniotomy Does Have its Share in the Management of Chronic Subdural Hematoma. *Indian Journal of Neurotrauma.* 2018;15(2):57-61.
58. Kim JH, Kang DS, Kim JH, Kong MH, Song KY. Chronic subdural hematoma treated by small or large craniotomy with membranectomy as the initial treatment. *J Korean Neurosurg Soc.* 2011;50(2):103-108.
59. Okano A, Oya S, Fujisawa N, et al. Analysis of risk factors for chronic subdural haematoma recurrence after burr hole surgery: optimal management of patients on antiplatelet therapy. *Br J Neurosurg.* 2014;28(2):204-208.
60. Goto H, Ishikawa O, Nomura M, Tanaka K, Nomura S, Maeda K. Magnetic resonance imaging findings predict the recurrence of chronic subdural hematoma. *Neurol Med Chir (Tokyo).* 2015;55(2):173-178.
61. Weng W, Li H, Zhao X, et al. The depth of catheter in chronic subdural haematoma: does it matter? *Brain Injury.* 2019;33(6):717-722.
62. Singh AK, Suryanarayanan B, Choudhary A, Prasad A, Singh S, Gupta LN. A prospective randomized study of use of drain versus no drain after burr-hole evacuation of chronic subdural hematoma. *Neurology India.* 2014;62(2):169-174.
63. Wu Q, Liu Q, Chen D, et al. Subdural drainage techniques for single burr-hole evacuation of chronic subdural hematoma: two drains frontal-occipital position versus one drain frontal position. *Br J Neurosurg.* 2021;35(3):324-328.
64. Flint AC, Chan SL, Rao VA, Efron AD, Kalani MA, Sheridan WF. Treatment of chronic subdural hematomas with subdural evacuating port system placement in the intensive care unit: evolution of practice and comparison with bur hole evacuation in the operating room. *J Neurosurg.* 2017;127(6):1443-1448.
65. Fujisawa N, Oya S, Yoshida S, et al. A Prospective Randomized Study on the Preventive Effect of Japanese Herbal Kampo Medicine Goreisan for Recurrence of Chronic Subdural Hematoma. *Neurol Med Chir (Tokyo).* 2021;61(1):12-20.
66. Adachi A, Higuchi Y, Fujikawa A, et al. Risk factors in chronic subdural hematoma: comparison of irrigation with artificial cerebrospinal fluid and normal saline in a cohort analysis. *PLoS One.* 2014;9(8):e103703.
67. Santarius T, Kirkpatrick PJ, Ganesan D, et al. Use of drains versus no drains after burr-hole evacuation of chronic subdural haematoma: a randomised controlled trial. *The Lancet.* 2009;374(9695):1067-1073.
68. Tsai TH, Lieu AS, Hwang SL, Huang TY, Hwang YF. A comparative study of the patients with bilateral or unilateral chronic subdural hematoma: precipitating factors and postoperative outcomes. *Journal of Trauma-Injury Infection & Critical Care.* 2010;68(3):571-575.
69. Hsieh CT, Su IC, Hsu SK, Huang CT, Lian FJ, Chang CJ. Chronic subdural hematoma: Differences between unilateral and bilateral occurrence. *J Clin Neurosci.* 2016;34:252-258.
70. Li F, Hua C, Feng Y, Yuan H, Bie L. Correlation of vascular endothelial growth factor with magnetic resonance imaging in chronic subdural hematomas. *J Neurol Sci.* 2017;377:149-154.
71. Mori K, Maeda M. Surgical treatment of chronic subdural hematoma in 500 consecutive cases: clinical characteristics, surgical outcome, complications, and recurrence rate. *Neurol Med Chir (Tokyo).* 2001;41(8):371-381.
72. Jang KM, Choi HH, Mun HY, Nam TK, Park YS, Kwon JT. Critical Depressed Brain Volume Influences the Recurrence of Chronic Subdural Hematoma after Surgical Evacuation. *Sci Rep.* 2020;10(1):1145.

73. Jang KM, Kwon JT, Hwang SN, Park YS, Nam TK. Comparison of the Outcomes and Recurrence with Three Surgical Techniques for Chronic Subdural Hematoma: Single, Double Burr Hole, and Double Burr Hole Drainage with Irrigation. *Korean J Neurotrauma.* 2015;11(2):75-80.
74. Zakaraia AM, Adnan JS, Haspani MS, Naing NN, Abdullah JM. Outcome of 2 different types of operative techniques practiced for chronic subdural hematoma in Malaysia: an analysis. *Surgical Neurology.* 2008;69(6):608-615; discussion 616.
75. Hamilton MG, Frizzell JB, Tranmer BI. Chronic subdural hematoma: the role for craniotomy reevaluated. *Neurosurgery.* 1993;33(1):67-72.
76. Yamamoto H, Hirashima Y, Hamada H, Hayashi N, Origasa H, Endo S. Independent predictors of recurrence of chronic subdural hematoma: results of multivariate analysis performed using a logistic regression model. *J Neurosurg.* 2003;98(6):1217-1221.
77. Abouzari M, Rashidi A, Rezaii J, et al. The role of postoperative patient posture in the recurrence of traumatic chronic subdural hematoma after burr-hole surgery. *Neurosurgery.* 2007;61(4):794-797; discussion 797.
78. Eggert HR, Harders A, Weigel K, Gilsbach J. Relapses after burr-hole drainage of chronic subdural haematomas. *Neurochirurgia.* 1984;27(5):141-143.
79. Yamada SM, Tomita Y, Murakami H, et al. Headache in patients with chronic subdural hematoma: analysis in 1080 patients. *Neurosurgical Review.* 2018;41(2):549-556.
80. Kareem H, Adams H. A closed system irrigation & drainage technique for surgical evacuation of chronic subdural haematomas. *F1000Res.* 2018;7:619.
81. Kuroki T, Katsume M, Harada N, Yamazaki T, Aoki K, Takasu N. Strict closed-system drainage for treating chronic subdural haematoma. *Acta Neurochir (Wien).* 2001;143(10):1041-1044.
82. Wang K, Chen D, Cao X, Gao L. A Prospective Comparative Study of Twist Drill Craniostomy Versus Burr Hole Craniostomy in Patients with Chronic Subdural Hematoma. *Turkish Neurosurgery.* 2017;27(1):60-65.
83. Ak H, Gülşen İ, Yaycioğlu S, et al. The effects of membranous abnormalities on mortality and morbidity in chronic subdural hematomas. *Journal of Neurological Sciences.* 2015;32(1):154-160.
84. Ishfaq A. Outcome in Chronic Subdural Hematoma After Subdural vs. Subgaleal Drain. *J Coll Physicians Surg Pak.* 2017;27(7):419-422.
85. Toi H, Fujii Y, Iwama T, et al. Determining if Cerebrospinal Fluid Prevents Recurrence of Chronic Subdural Hematoma: A Multi-Center Prospective Randomized Clinical Trial. *Journal of Neurotrauma.* 2019;36(4):559-564.
86. Chang CL, Sim JL, Delgardo MW, Ruan DT, Connolly ES, Jr. Predicting Chronic Subdural Hematoma Resolution and Time to Resolution Following Surgical Evacuation. *Front Neurol.* 2020;11:677.
87. Blaauw J, Jacobs B, den Hertog HM, et al. Neurosurgical and Perioperative Management of Chronic Subdural Hematoma. *Front Neurol.* 2020;11:550.
88. Morales-Gomez JA, Garcia-Estrada E, Garza-Baez A, Mercado-Flores M, de Leon AM. Subdural open drains as an effective and low-cost modality for the treatment of chronic subdural hematomas. *Br J Neurosurg.* 2020:1-4.
89. Ryu SM, Yeon JY, Kong DS, Hong SC. Risk of Recurrent Chronic Subdural Hematoma Associated with Early Warfarin Resumption: A Matched Cohort Study. *World Neurosurgery.* 2018;120:e855-e862.
90. Lin X. Comparing twist-drill drainage with burr hole drainage for chronic subdural hematoma. *Chinese Journal of Traumatology.* 2011;14(3):170-173.
91. Rovlias A, Theodoropoulos S, Papoutsakis D. Chronic subdural hematoma: Surgical management and outcome in 986 cases: A classification and regression tree approach. *Surgical neurology international.* 2015;6:127.
92. Cheng SY, Chang CK, Chen SJ, Lin JF, Tsai CC. Chronic subdural hematoma in elderly Taiwan patients: A retrospective analysis of 342 surgical cases. *International Journal of Gerontology.* 2014;8(1):37-41.

93. Shah S, Rehman L, Ahmed N, Chaudhry MA, Shabir A. Comparison of recurrence of chronic subdural haematoma after burr hole craniostomy with one time drainage and burr hole craniostomy with tube drainage. *Pakistan Journal of Medical and Health Sciences.* 2014;8(4):1027-1029.
94. Gabarros A, Acebes JJ, Rodriguez R, et al. Results of surgical treatment in chronic subdural hematoma. Comparison between two technique: Twist-drill and continuous closed drainage versus two burr holes and open external drainage. *Neurocirugia.* 2000;11(5):377-390.
95. Tanikawa M, Mase M, Yamada K, et al. Surgical treatment of chronic subdural hematoma based on intrahematomal membrane structure on MRI. *Acta Neurochirurgica.* 2001;143(6):613-618; discussion 618-619.
96. Motoie R, Karashima S, Otsuji R, et al. Recurrence in 787 Patients with Chronic Subdural Hematoma: Retrospective Cohort Investigation of Associated Factors Including Direct Oral Anticoagulant Use. *World Neurosurgery.* 2018;118:e87-e91.
97. Zhang JJY, Wang S, Foo ASC, et al. Outcomes of Subdural Versus Subperiosteal Drain After Burr-Hole Evacuation of Chronic Subdural Hematoma: A Multicenter Cohort Study. *World Neurosurgery.* 2019;131:e392-e401.
98. Bankole OB, Yusuf AS, Kanu OO, Ukponmwan E, Nnadi MN, Arigbabu SO. Chronic subdural haematoma: Clinical presentation, surgical treatment and outcome at the lagos university teaching hospital. *African Journal of Neurological Sciences.* 2011;30(1).
99. ShafiqAlam S, UdDin MA, Sheikh JI, et al. Chronic subdural hematoma-an experience of 432 cases in a tertiary care centre. *JK Practitioner.* 2017;22(1):20-24.
100. Katayama K, Matsuda N, Kakuta K, et al. The Effect of Goreisan on the Prevention of Chronic Subdural Hematoma Recurrence: Multi-Center Randomized Controlled Study. *J Neurotrauma.* 2018;35(13):1537-1542.
101. Kim SU, Lee DH, Kim YI, Yang SH, Sung JH, Cho CB. Predictive Factors for Recurrence after Burr-Hole Craniostomy of Chronic Subdural Hematoma. *J Korean Neurosurg Soc.* 2017;60(6):701-709.
102. Castro-Rodriguez C, Roman-Pena P, Aran-Echabe E, Gelabert-Gonzalez M. [Chronic subdural haematomas in very elderly patients]. *Revista Espanola de Geriatria y Gerontologia.* 2016;51(6):309-316.
103. Tagle P, Mery F, Torrealba G, et al. [Chronic subdural hematoma: a disease of elderly people]. *Rev Med Chil.* 2003;131(2):177-182.
104. Nunta-Aree S, Paruang T, Sitthinamsuwan B. Timing of brain expansion and recurrence after surgery of chronic subdural hematoma. *Journal of the Medical Association of Thailand.* 2017;100(4):S59-S64.
105. Shim YW, Lee WH, Lee KS, Kim ST, Paeng SH, Pyo SY. Burr Hole Drainage versus Small Craniotomy of Chronic Subdural Hematomas. *Korean Journal of Neurotrauma.* 2019;15(2):110-116.
106. Sjavik K, Bartek J, Jr., Sagberg LM, et al. Assessment of drainage techniques for evacuation of chronic subdural hematoma: a consecutive population-based comparative cohort study. *Journal of Neurosurgery.* 2017:1-7.
107. Yan K, Gao H, Zhou X, et al. A retrospective analysis of postoperative recurrence of septated chronic subdural haematoma: endoscopic surgery versus burr hole craniotomy. *Neurological Research.* 2017;39(9):803-812.
108. Bartek J, Jr., Sjavik K, Stahl F, et al. Surgery for chronic subdural hematoma in nonagenarians: A Scandinavian population-based multicenter study. *Acta Neurol Scand.* 2017;136(5):516-520.
109. Ram Z, Hadani M, Sahar A, Spiegelmann R. Continuous irrigation-drainage of the subdural space for the treatment of chronic subdural haematoma. A prospective clinical trial. *Acta Neurochirurgica.* 1993;120(1):40-43.

110. Baechli H, Nordmann A, Bucher HC, Gratzl O. Demographics and prevalent risk factors of chronic subdural haematoma: results of a large single-center cohort study. *Neurosurg Rev.* 2004;27(4):263-266.
111. Jung YG, Jung NY, Kim E. Independent predictors for recurrence of chronic subdural hematoma. *J Korean Neurosurg Soc.* 2015;57(4):266-270.
112. Dobran M, Marini A, Nasi D, et al. Clinical Outcome of Patients Over 90 Years of Age Treated for Chronic Subdural Hematoma. *J Korean Neurosurg Soc.* 2022;65(1):123-129.
113. Carlsen JG, Cortnum S, Sorensen JC. Recurrence of chronic subdural haematomata with and without post-operative drainage. *Br J Neurosurg.* 2011;25(3):388-390.
114. Erol FS, Topsakal C, Faik Ozveren M, Kaplan M, Tiftikci MT. Irrigation vs. closed drainage in the treatment of chronic subdural hematoma. *J Clin Neurosci.* 2005;12(3):261-263.
115. Frati A, Salvati M, Mainiero F, et al. Inflammation markers and risk factors for recurrence in 35 patients with a posttraumatic chronic subdural hematoma: a prospective study. *J Neurosurg.* 2004;100(1):24-32.
116. Huang YH, Yang KY, Lee TC, Liao CC. Bilateral chronic subdural hematoma: what is the clinical significance? *Int J Surg.* 2013;11(7):544-548.
117. Lo WL, Lee TC, Fang PS, Huang YH. Chronic subdural hematoma in patients under age 65 years: A comparative study of age cohort. *Formosan Journal of Surgery.* 2013;46(1):10-14.
118. Yan C, Yang MF, Huang YW. A Reliable Nomogram Model to Predict the Recurrence of Chronic Subdural Hematoma After Burr Hole Surgery. *World Neurosurgery.* 2018;118:e356-e366.
119. Gonugunta V, Buxton N. Warfarin and chronic subdural haematomas. *Br J Neurosurg.* 2001;15(6):514-517.
120. Wang N, Hu J, Oppong-Gyebi A, et al. Elevated blood urea nitrogen is associated with recurrence of post-operative chronic subdural hematoma. *BMC Neurol.* 2020;20(1):411.
121. Tugcu B, Tanriverdi O, Baydin S, et al. Can recurrence of chronic subdural hematoma be predicted? A retrospective analysis of 292 cases. *Journal of Neurological Surgery.* 2014;75(1):37-41.
122. D'Oria S, Dibenedetto M, Squillante E, et al. Chronic subdural hematomas: single versus double burr holes. *J Neurosurg Sci.* 2020;64(2):216-218.
123. Zumofen D, Regli L, Levivier M, Krayenbuhl N. Chronic subdural hematomas treated by burr hole trepanation and a subperiostal drainage system. *Neurosurgery.* 2009;64(6):1116-1121; discussion 1121-1112.
124. You W, Zhu Y, Wang Y, et al. Prevalence of and risk factors for recurrence of chronic subdural hematoma. *Acta Neurochirurgica.* 2018;160(5):893-899.
125. Shen J, Yuan L, Ge R, et al. Clinical and radiological factors predicting recurrence of chronic subdural hematoma: A retrospective cohort study. *Injury.* 2019;50(10):1634-1640.
126. Sarnvivad P, Chiewchanvechakul W, Chumnanvej S. Chronic subdural hematoma: drainage vs. no drainage. *Journal of the Medical Association of Thailand.* 2011;94(11):1352-1356.
127. Janowski M, Kunert P. Intravenous fluid administration may improve post-operative course of patients with chronic subdural hematoma: a retrospective study. *PLoS One.* 2012;7(4):e35634.
128. Heringer LC, Sousa UO, Oliveira MF, et al. The number of burr holes and use of a drain do not interfere with surgical results of chronic subdural hematomas. *Arq Neuropsiquiatr.* 2017;75(11):809-812.
129. Raghavan A, Smith G, Onyewadume L, et al. Morbidity and Mortality After Burr Hole Craniostomy Versus Craniotomy for Chronic Subdural Hematoma Evacuation: A Single-Center Experience. *World Neurosurgery.* 2020;134:e196-e203.
130. Lee JY, Ebel H, Ernestus RI, Klug N. Various surgical treatments of chronic subdural hematoma and outcome in 172 patients: is membranectomy necessary? *Surgical Neurology.* 2004;61(6):523-527; discussion 527-528.

131. Maldaner N, Sosnova M, Sarnthein J, Bozinov O, Regli L, Stienen MN. Predicting Functional Impairment in patients with chronic subdural hematoma treated with burr hole Trepanation-The FIT-score. *Clinical Neurology & Neurosurgery.* 2019;182:142-147.
132. Poulsen FR, Munthe S, Soe M, Halle B. Perindopril and residual chronic subdural hematoma volumes six weeks after burr hole surgery: a randomized trial. *Clin Neurol Neurosurg.* 2014;123:4-8.
133. Stanisic M, Hald J, Rasmussen IA, et al. Volume and densities of chronic subdural haematoma obtained from CT imaging as predictors of postoperative recurrence: a prospective study of 107 operated patients. *Acta Neurochirurgica.* 2013;155(2):323-333; discussion 333.
134. Bellut D, Woernle CM, Burkhardt JK, Kockro RA, Bertalanffy H, Krayenbuhl N. Subdural drainage versus subperiosteal drainage in burr-hole trepanation for symptomatic chronic subdural hematomas. *World Neurosurg.* 2012;77(1):111-118.
135. Ahmed AU, Tyler MA, Thaci B, et al. A comparative study of neural and mesenchymal stem cell-based carriers for oncolytic adenovirus in a model of malignant glioma. *Mol Pharm.* 2011;8(5):1559-1572.
136. Eppel M. SI, Gorzer H., Ferraz-Leite H. Head injury and chronic subdural hematoma. *Acta Chirurgica Austriaca.* 1999;31.
137. Qian Z, Yang D, Sun F, Sun Z. Risk factors for recurrence of chronic subdural hematoma after burr hole surgery: potential protective role of dexamethasone. *Br J Neurosurg.* 2017;31(1):84-88.
138. Hori YS, Ebisudani Y, Aoi M, Fukuhara T. Elevated Serum Fibrinogen Degradation Products on Admission Is a Novel Predictive Factor for Recurrence of Chronic Subdural Hematoma. *World Neurosurg.* 2018;118:e753-e757.
139. Song DH, Kim YS, Chun HJ, et al. The Predicting Factors for Recurrence of Chronic Subdural Hematoma Treated with Burr Hole and Drainage. *Korean Journal of Neurotrauma.* 2014;10(2):41-48.
140. Kocaman U, Yilmaz H. Description of a Modified Technique (mini craniotomy-basal membranotomy) for Chronic Subdural Hematoma Surgery and Evaluation of the Contribution of Basal Membranotomy Performed as Part of This Technique to Cerebral Expansion. *World Neurosurg.* 2019;122:e1002-e1006.
141. Lee J, Park JH. Clinical Characteristics of Bilateral versus Unilateral Chronic Subdural Hematoma. *Korean J Neurotrauma.* 2014;10(2):49-54.
142. Wang QF, Cheng C, You C. A New Modified Twist Drill Craniostomy Using a Novel Device to Evacuate Chronic Subdural Hematoma. *Medicine.* 2016;95(10):e3036.
143. Gilsbach J, Eggert HR, Harders A. External closed drainage treatment of chronic subdural hematomas after bore-hole trepanation. *Unfallchirurgie.* 1980;6(3):183-186.
144. Iftikhar M, Siddiqui UT, Rauf MY, Malik AO, Javed G. Comparison of Irrigation versus No Irrigation during Burr Hole Evacuation of Chronic Subdural Hematoma. *J Neurol Surg A Cent Eur Neurosurg.* 2016;77(5):416-421.
145. Klein J, Mauck L, Schackert G, Pinzer T. Do statins reduce the rate of revision surgery after chronic subdural hematoma drain? *Acta Neurochir (Wien).* 2021;163(7):1843-1848.
146. Miki K, Abe H, Morishita T, et al. Double-crescent sign as a predictor of chronic subdural hematoma recurrence following burr-hole surgery. *Journal of Neurosurgery.* 2019;131(6):1905-1911.
147. Ernestus RI, Beldzinski P, Lanfermann H, Klug N. Chronic subdural hematoma: surgical treatment and outcome in 104 patients. *Surg Neurol.* 1997;48(3):220-225.
148. Torihashi K, Sadamasa N, Yoshida K, Narumi O, Chin M, Yamagata S. Independent predictors for recurrence of chronic subdural hematoma: a review of 343 consecutive surgical cases. *Neurosurgery.* 2008;63(6):1125-1129; discussion 1129.
149. Schwarz F, Loos F, Dunisch P, et al. Risk factors for reoperation after initial burr hole trephination in chronic subdural hematomas. *Clinical Neurology & Neurosurgery.* 2015;138:66-71.

150. Nakagawa I, Park HS, Kotsugi M, et al. Enhanced Hematoma Membrane on DynaCT Images During Middle Meningeal Artery Embolization for Persistently Recurrent Chronic Subdural Hematoma. *World Neurosurg.* 2019.
151. Ridwan S, Bohrer AM, Grote A, Simon M. Surgical Treatment of Chronic Subdural Hematoma: Predicting Recurrence and Cure. *World Neurosurgery.* 2019;128:e1010-e1023.
152. Drapkin AJ. Chronic subdural hematoma: pathophysiological basis for treatment. *Br J Neurosurg.* 1991;5(5):467-473.
153. Martinez-Perez R, Tsimpas A, Rayo N, Cepeda S, Lagares A. Role of the patient comorbidity in the recurrence of chronic subdural hematomas. *Neurosurgical Review.* 2020;7:07.
154. Abboud T, Duhrsen L, Gibbert C, Westphal M, Martens T. Influence of antithrombotic agents on recurrence rate and clinical outcome in patients operated for chronic subdural hematoma. *Neurocirugia (Astur).* 2018;29(2):86-92.
155. Gurelik M, Aslan A, Gurelik B, Ozum U, Karadag O, Kars HZ. A safe and effective method for treatment of chronic subdural haematoma. *Can J Neurol Sci.* 2007;34(1):84-87.
156. Choi J, Pyen J, Cho S, Kim J, Koo Y, Whang K. Influence of Antithrombotic Medication on the Risk of Chronic Subdural Hematoma Recurrence after Burr-Hole Surgery. *J Korean Neurosurg Soc.* 2020;63(4):513-518.
157. Edem I, Moldovan ID, Turner A, Alkherayf F. A comparative study of chronic subdural hematoma Burr hole craniostomy treatment: To irrigate or not to irrigate. *Interdisciplinary Neurosurgery: Advanced Techniques and Case Management.* 2019;18.
158. Miah IP, Herklots M, Roks G, et al. Dexamethasone Therapy in Symptomatic Chronic Subdural Hematoma (DECSA-R): A Retrospective Evaluation of Initial Corticosteroid Therapy versus Primary Surgery. *J Neurotrauma.* 2020;37(2):366-372.
159. Lee JK, Choi JH, Kim CH, Lee HK, Moon JG. Chronic subdural hematomas : a comparative study of three types of operative procedures. *J Korean Neurosurg Soc.* 2009;46(3):210-214.
160. Chon KH, Lee JM, Koh EJ, Choi HY. Independent predictors for recurrence of chronic subdural hematoma. *Acta Neurochir (Wien).* 2012;154(9):1541-1548.
161. Schoedel P, Bruendl E, Hochreiter A, et al. Restoration of Functional Integrity After Evacuation of Chronic Subdural Hematoma-An Age-Adjusted Analysis of 697 Patients. *World Neurosurgery.* 2016;94:465-470.
162. Munoz-Bendix C, Pannewitz R, Remmel D, et al. Outcome following surgical treatment of chronic subdural hematoma in the oldest-old population. *Neurosurg Rev.* 2017;40(3):461-468.
163. Kristof RA, Grimm JM, Stoffel-Wagner B. Cerebrospinal fluid leakage into the subdural space: possible influence on the pathogenesis and recurrence frequency of chronic subdural hematoma and subdural hygroma. *J Neurosurg.* 2008;108(2):275-280.
164. Won SY, Dubinski D, Behmanesh B, et al. Supervised Valsalva Maneuver after Burr Hole Evacuation of Chronic Subdural Hematomas: A Prospective Cohort Study. *J Neurotrauma.* 2021;38(7):911-917.
165. Hani L, Vulcu S, Branca M, et al. Subdural versus subgaleal drainage for chronic subdural hematomas: a post hoc analysis of the TOSCAN trial. *J Neurosurg.* 2019:1-9.
166. Khan HU, Atif K, Boghsani GT. Single versus double burr-hole drainage for chronic subdural hematoma: A study of relevant prognostic factors conducted in Pakistan. *Pak J Med Sci.* 2019;35(4):963-968.
167. Harders A, Eggert HR, Weigel K. [Treatment of chronic subdural haematoma by closed external drainage]. *Neurochirurgia (Stuttg).* 1982;25(5):147-152.
168. Kim DH, Kim HS, Choi HJ, Han IH, Cho WH, Nam KH. Recurrence of the Chronic Subdural Hematoma after Burr-Hole Drainage with or without Intraoperative Saline Irrigation. *Korean J Neurotrauma.* 2014;10(2):101-105.
169. Krupp WF, Jans PJ. Treatment of chronic subdural haematoma with burr-hole craniostomy and closed drainage. *Br J Neurosurg.* 1995;9(5):619-627.

170. Tahsim-Oglou Y, Beseoglu K, Hanggi D, Stummer W, Steiger HJ. Factors predicting recurrence of chronic subdural haematoma: the influence of intraoperative irrigation and low-molecular-weight heparin thromboprophylaxis. *Acta Neurochirurgica.* 2012;154(6):1063-1067; discussion 1068.
171. Zhang J, Liu X, Fan X, et al. The use of endoscopic-assisted burr-hole craniostomy for septated chronic subdural haematoma: A retrospective cohort comparison study. *Brain Research.* 2018;1678:245-253.
172. Kim HC, Ko JH, Yoo DS, Lee SK. Spontaneous Resolution of Chronic Subdural Hematoma : Close Observation as a Treatment Strategy. *J Korean Neurosurg Soc.* 2016;59(6):628-636.
173. Rohde V, Graf G, Hassler W. Complications of burr-hole craniostomy and closed-system drainage for chronic subdural hematomas: a retrospective analysis of 376 patients. *Neurosurg Rev.* 2002;25(1-2):89-94.
174. Wan Y, Fei X, Jiang D, Chen H, Shi L, Wang Z. Clinical Observation of Treatment of Chronic Subdural Hematoma With Novel Double Needle Minimally Invasive Aspiration Technology. *Journal of Craniofacial Surgery.* 2017;28(3):646-649.
175. Gernsback J, Kolcun JP, Jagid J. To Drain or Two Drains: Recurrences in Chronic Subdural Hematomas. *World Neurosurgery.* 2016;95:447-450.
176. Chen S, Chen Z, Yang B, Xu T, Tu XK. Use of siphon irrigation during burr-hole craniostomy to evacuate chronic subdural hematoma: A retrospective cohort comparison study. *Medicine (Baltimore).* 2020;99(21):e20291.
177. Yamada T, Natori Y. Prospective Study on the Efficacy of Orally Administered Tranexamic Acid and Goreisan for the Prevention of Recurrence After Chronic Subdural Hematoma Burr Hole Surgery. *World Neurosurgery.* 2020;134:e549-e553.
178. Hennig R, Kloster R. Burr hole evacuation of chronic subdural haematomas followed by continuous inflow and outflow irrigation. *Acta Neurochir (Wien).* 1999;141(2):171-176.
179. Kale A, Oz, II, Gun EG, Kalayci M, Gul S. Is the recurrence rate of chronic subdural hematomas dependent on the duration of drainage? *Neurological Research.* 2017;39(5):399-402.
180. Kaminogo M, Moroki J, Ochi A, et al. Characteristics of symptomatic chronic subdural haematomas on high-field MRI. *Neuroradiology.* 1999;41(2):109-116.
181. Hirai S, Yagi K, Hara K, Kanda E, Matsubara S, Uno M. Postoperative recurrence of chronic subdural hematoma is more frequent in patients with blood type A. *J Neurosurg.* 2021:1-5.
182. Tosaka M, Tsushima Y, Watanabe S, et al. Superficial subarachnoid cerebrospinal fluid space expansion after surgical drainage of chronic subdural hematoma. *Acta Neurochirurgica.* 2015;157(7):1205-1214.
183. Takei J, Hirotsu T, Hatano K, et al. Modified Computed Tomography Classification for Chronic Subdural Hematoma Features Good Interrater Agreement: A Single-Center Retrospective Cohort Study. *World Neurosurg.* 2021;151:e407-e417.
184. Weigel R, Schlickum L, Weisser G, Krauss JK. Treatment concept of chronic subdural haematoma according to an algorithm using evidence-based medicine-derived key factors: A prospective controlled study. *British Journal of Neurosurgery.* 2015;29(4):538-543.
185. Mellergard P, Wisten O. Operations and re-operations for chronic subdural haematomas during a 25-year period in a well defined population. *Acta Neurochir (Wien).* 1996;138(6):708-713.
186. Taussky P, Fandino J, Landolt H. Number of burr holes as independent predictor of postoperative recurrence in chronic subdural haematoma. *British Journal of Neurosurgery.* 2008;22(2):279-282.
187. Amano T, Matsuo S, Miyamatsu Y, Yamashita S, Nakamizo A. Impact of antithrombotic therapy on surgical treatment in patients with chronic subdural hematoma. *J Clin Neurosci.* 2020;74:55-60.
188. Nakaguchi H, Tanishima T, Yoshimasu N. Factors in the natural history of chronic subdural hematomas that influence their postoperative recurrence. *J Neurosurg.* 2001;95(2):256-262.

189. Borger V, Vatter H, Oszvald A, Marquardt G, Seifert V, Guresir E. Chronic subdural haematoma in elderly patients: a retrospective analysis of 322 patients between the ages of 65-94 years. *Acta Neurochir (Wien).* 2012;154(9):1549-1554.
