# Supplementary material for: Recurrent Chronic Subdural Hematoma After Burr-Hole Surgery and Postoperative Drainage: A Systematic Review and Meta-Analysis
Source: Oper Neurosurg. 2023 Jun 30;25(3):216–41. doi: 10.1227/ons.0000000000000794 (PMC10389757; doi:10.1227/ons.0000000000000794)
Supplement: Supplementary file 4 [file ons-25-216-s004.pdf]

Supplementary

Table 1. Syntax search

Medline (ovid) search 1			Embase search 1			Medline (ovid) search 2			Embase search 2		
No.	Query	Results	No.	Query	Results	No.	Query	Results	No.	Query	Results
1	exp Hematoma, Subdural, Chronic/	1302	#11	#9 NOT #10	1865	1	exp Hematoma, Subdural, Chronic/	1446	#11	#9 NOT #10	2162
2	((Subdural adj (H?ematoma or Hemorrhage)) or CSDH).ti,ab,kf.	7934	#10	#8 AND [embase]/lim AND 'Conference Abstract'/it	294	2	((Subdural adj (H?ematoma or Hemorrhage)) or CSDH).ti,ab,kf.	8584	#10	#8 AND [embase]/lim AND 'Conference Abstract'/it	327
3	exp Drainage/	58453	#9	#8 AND [embase]/lim	2159	3	exp Drainage/	60970	#9	#8 AND [embase]/lim	2489
4	(Drain* or Burr-Hole* or Burrhole* or craniostom*).ti,ab,kf.	130714	#8	#3 AND #7	2375	4	(Drain* or Burr-Hole* or Burrhole* or craniostom*).ti,ab,kf.	140135	#8	#3 AND #7	2741
5	Trepan*.ti,ab,kf.	972	#7	#4 OR #5 OR #6	243393	5	Trepan*.ti,ab,kf.	1023	#7	#4 OR #5 OR #6	263813
6	Trephin*.ti,ab,kf.	4077	#6	'surgical drainage'/exp OR 'cerebrospinal fluid drainage'/exp	87330	6	Trephin*.ti,ab,kf.	4256	#6	'surgical drainage'/exp OR 'cerebrospinal fluid drainage'/exp	93641

7	Trephining/	1505	#5	drain*:ti,ab,de,kw OR 'burr hole*':ti,ab,de,kw OR burrhole*:ti,ab,de,kw OR craniostom*:ti,ab,de,kw OR trepan*:ti,ab,de,kw OR trephin*:ti,ab,de,kw	233395	7	Trephining/	1569	#5	drain*:ti,ab,de,kw OR 'burr hole*':ti,ab,de,kw OR burrhole*:ti,ab,de,kw OR craniostom*:ti,ab,de,kw OR trepan*:ti,ab,de,kw OR trephin*:ti,ab,de,kw	252791
8	3 or 4 or 5 or 6 or 7	166107	#4	'burr hole'/exp	39	8	3 or 4 or 5 or 6 or 7	176739	#4	'burr hole'/exp	43
9	1 or 2	8077	#3	#1 OR #2	18300	9	1 or 2	8734	#3	#1 OR #2	21067
10	8 and 9	1514	#2	((subdural NEAR/1 (h?ematoma OR hemorrhage)):ti,ab,kw) OR csdh:ti,ab,kw	3653	10	8 and 9	1654	#2	((subdural NEAR/1 (h?ematoma OR hemorrhage)):ti,ab,kw) OR csdh:ti,ab,kw	4067
11	exp animals/ not humans/	4678989	#1	'subdural hematoma'/exp	17808	11	exp animals/ not humans/	4837305	#1	'subdural hematoma'/exp OR 'subdural hematoma'	20602

12	10 not 11	1512				12	10 not 11	1651			
