# Supplementary material for: Recurrent Chronic Subdural Hematoma After Burr-Hole Surgery and Postoperative Drainage: A Systematic Review and Meta-Analysis
Source: Oper Neurosurg. 2023 Jun 30;25(3):216–41. doi: 10.1227/ons.0000000000000794 (PMC10389757; doi:10.1227/ons.0000000000000794)
Supplement: Supplementary file 5 [file ons-25-216-s005.pdf]

Supplementary Table 2. Pooled incidence and recurrence rate of irrigation methods in studies using a definition of clinical and radiological factors and a reoperation.

Irrigation method	Number of studies	Number of patients	Pooled incidence	Recurrence rate[@]
Saline/normal saline/isotonic saline/physiological saline	21	4157	61.1%	12.5%
Ringer solution/Hartmann's solution	3	333	4.9%	16.5%
Gentamicine induced irrigation method	2	622	9.1%	11.4%
Warm saline	10	1272	18.7%	11.8%
Warm Ringer solution	3	421	6.2%	12.6%

[@] Calculated by dividing number of patient per group by the total number of patients in which irrigation method was described
