# Supplementary material for: Recurrent Chronic Subdural Hematoma After Burr-Hole Surgery and Postoperative Drainage: A Systematic Review and Meta-Analysis
Source: Oper Neurosurg. 2023 Jun 30;25(3):216–41. doi: 10.1227/ons.0000000000000794 (PMC10389757; doi:10.1227/ons.0000000000000794)
Supplement: Supplementary file 6 [file ons-25-216-s006.pdf]

Supplementary Table 3. Pooled incidence and recurrence rate of postoperative drain location used in studies using a definition of clinical and radiological factors and a reoperation.

Location of postoperative drain	Number of studies	Number of patients	Pooled incidence	Recurrence rate[@]
Subdural	44	7573	98.1%	13.5%
Subperiostal	1	147	1.9%	15%
Subgaleal	No studies			

[@] Calculated by dividing the number of patients per group by the total number of patients in which postoperative drain location was described
