# Supplementary material for: Recurrent Chronic Subdural Hematoma After Burr-Hole Surgery and Postoperative Drainage: A Systematic Review and Meta-Analysis
Source: Oper Neurosurg. 2023 Jun 30;25(3):216–41. doi: 10.1227/ons.0000000000000794 (PMC10389757; doi:10.1227/ons.0000000000000794)
Supplement: Supplementary file 7 [file ons-25-216-s007.pdf]

Supplementary Table 4. Pooled incidence and recurrence rate of postoperative drainage time used in studies using a definition of clinical and radiological factors and a reoperation.

Drainage time in hours*	Number of studies	Number of patients	Pooled incidence	Recurrence rate[@]
≤24h	3	315	3.9%	12.4%
≤48h	17	5299	66.0%	12.9%
≤72h	11	1659	20.7%	17.1%
≤96h	2	137	1.7%	17.5%
≤120h	4	617	7.7%	10.2%

[@] Calculated by dividing the number of patients per group by the total number of patients in which postoperative drainage time was described
